# Implementation of the CRISPR-Cas13a system in fission yeast and its repurposing for precise RNA editing

**DOI:** 10.1093/nar/gky433

**Published:** 2018-05-31

**Authors:** Xinyun Jing, Bingran Xie, Longxian Chen, Niubing Zhang, Yiyi Jiang, Hang Qin, Hongbing Wang, Pei Hao, Sheng Yang, Xuan Li

**Affiliations:** 1 Key Laboratory of Synthetic Biology, CAS Center for Excellence in Molecular Plant Sciences, Institute of Plant Physiology and Ecology, Chinese Academy of Sciences, Shanghai 200032, China; 2 School of Life Sciences, Henan University, Kaifeng 475000, China; 3 Key Laboratory of Molecular Virology and Immunology, Institut Pasteur of Shanghai, Chinese Academy of Sciences, Shanghai 200031, China; 4 Department of Physiology, Michigan State University, East Lansing, Michigan, United States of America

## Abstract

In contrast to genome editing, which introduces genetic changes at the DNA level, disrupting or editing gene transcripts provides a distinct approach to perturbing a genetic system, offering benefits complementary to classic genetic approaches. To develop a new toolset for manipulating RNA, we first implemented a member of the type VI CRISPR systems, Cas13a from *Leptotrichia shahii* (LshCas13a), in *Schizosaccharomyces pombe*, an important model organism employed by biologists to study key cellular mechanisms conserved from yeast to humans. This approach was shown to knock down targeted endogenous gene transcripts with different efficiencies. Second, we engineered an RNA editing system by tethering an inactive form of LshCas13a (dCas13) to the catalytic domain of human adenosine deaminase acting on RNA type 2 (hADAR2d), which was shown to be programmable with crRNA to target messenger RNAs and precisely edit specific nucleotide residues. We optimized system parameters using a dual-fluorescence reporter and demonstrated the utility of the system in editing randomly selected endogenous gene transcripts. We further used it to restore the transposition of retrotransposon Tf1 mutants in fission yeast, providing a potential novel toolset for retrovirus manipulation and interference.

## INTRODUCTION

Modulation of gene expression or alteration of gene transcripts provides a critical layer of regulation at the RNA level in living cells. Editing or partially disrupting transcripts serves as a distinct approach for the analysis of gene functions, offering benefits complementary to classic genetic approaches and, possibly, novel insight into the regulation of gene function at the RNA level.

In recent years, many molecular tools utilizing CRISPR systems to disrupt genes or introduce coding changes at the DNA level have been successfully developed (1–3). From a different perspective, a synthetic tool for perturbing a genetic system by disrupting or editing gene transcripts is desirable. The discovery of type VI CRISPR systems (4–6) (e.g. members of the Cas13 family) has created an excellent opportunity to develop a new toolset for RNA manipulation experiments. Members of the Cas13 family display the unique ability to target single-strand RNA. They cleave RNA at sites guided by CRISPR RNA (crRNA) containing a variable-length spacer (7–10). One member of the Cas13 family, Cas13a from *Leptotrichia shahii* (LshCas13), was found to knockdown target transcripts in *Escherichia coli* and mammalian cells and has been used in nucleic acid detection and RNA tagging (7,8,11,12). More recently, another member of the Cas13 family, Cas13b from *Prevotella sp. P5-125*, was employed to construct a fusion protein for targeted editing of gene transcripts in mammalian cells, which was shown to correct disease-associated mutations in human cell lines (10).

The unicellular organism *Schizosaccharomyces pombe* (fission yeast) is a commonly employed, important model system used by biologists to study processes that are conserved from yeast to humans. The use of *S. pombe* in research has led to many key discoveries related to cell-cycle control (13,14), chromosome structure (15,16), histone modifications (17,18) and cytokinesis (19,20). Along the way, genetic manipulation tools have been developed and applied, with an exceptional scientific impact. To take advantage of the new advances in CRISPR research, in the current study, we set out to explore a synthetic toolset for manipulating gene transcripts in fission yeast by implementation and repurposing of the type VI CRISPR system.

There are unique advantages associated with the manipulation of transcripts instead of genes. Altering gene transcripts does not modify the genes themselves, making the changes reversible and more temporally and spatially controllable. This approach is also more efficient and effective when targeting genes with multiple copies, especially in polyploid organisms. It is a potentially useful alternative for disrupting or correcting mutated genes in diseased tissues for therapeutic purposes. In addition, the implementation of an RNA manipulation system in fission yeast has added benefits for studying cellular functions/processes because of the close similarity of these yeast to higher eukaryotic cells. There is no native protein similar to the adenosine deaminases acting on RNA (ADAR) family of enzymes (21) in fission yeast that would interfere with an engineered RNA editing system. Partially editing a gene’s transcripts allows one to simulate the effect of two different alleles for a given gene in haploid yeast strains. For genetic screening experiments in haploid fission yeast, conditional knockdown or editing of gene transcripts may circumvent the problem of lethal effects for some mutations and is easier to perform than traditional genetic methods.

Motivated by these goals, in the current study, we first sought to implement the LshCas13a system in *S. pombe* for targeting and disrupting gene transcripts. We then designed and engineered an RNA editing system by tethering an inactive form of LshCas13a (R1278A mutant) (dCas13a) to the catalytic domain of human adenosine deaminase acting on RNA 2 (hADAR2d). Similar to the mutant Cas9 variant (dCas9) that is capable of binding target DNA but inactive in DNA cleavage (22,23), the LshCas13a R1278A mutant (referred to as dCas13a) bound target RNA more strongly (*K*_D_ ∼ 7 nM) than the wild-type (*K*_D_ ∼ 46 nM), although it was catalytically inactive (7). Notably, the target RNA binding affinity of the R1278A mutant complex in the presence of crRNA was one order of magnitude higher than that of crRNA binding to target RNA (*K*_D_ ∼ 69 nM) (7). We showed *in vivo* that this fusion complex can be programmed to target gene transcripts and precisely edit specific nucleotide residues in the presence of crRNA. We optimized the system parameters and demonstrated the utility of the system in the editing of randomly selected endogenous gene transcripts, in addition to constructed fluorescent reporter transcripts. We further used this system to restore the transposition of retrotransposon Tf1 mutants in fission yeast. Our work introduces a new programmable toolset in fission yeast for transcriptomic manipulation that is widely applicable in basic genetic and biotechnological research.

## MATERIALS AND METHODS

### Plasmids and constructs

The plasmids and constructs employed in this work are listed in Supplementary Tables S1, 2, 4–7. The sequences of the oligonucleotides employed in the study are described in Dataset S1. Details of the construction of dCas13a expression vectors, dual-fluorescence reporter vectors, crRNA/pRNA expression vectors and retrotransposon Tf1 mutants are described in the Supplementary Methods.

Polymerase chain reaction (PCR) was performed using Taq (Thermo Fisher Scientific) or KOD FX DNA polymerase (TOYOBO). Plasmids and chromosomal DNA were extracted using the Plasmid Mini Kit I and Gel Extraction Kit from OMEGA. Cloning was performed using either restriction endonucleases and T4 DNA ligase (New England Biolabs) or the ClonExpress^®^ II One Step Cloning Kit (Vazyme). *Escherichia coli* DH5α was used for the purpose of molecular cloning.

### Strains and transformation

The *S. pombe* strain FY7652 containing the *ura4*-D18 and *leu1*-32 alleles was employed in this study (Supplementary Table S1). Yeast strains were grown in YES medium (24) supplemented with 50 μg/ml uracil and 50 μg/ml leucine at 30°C until reaching mid-log phase and then transformed using the Lithium Acetate/PEG/Heat shock method (24). For chromosomal expression of eGFP or dCas13a-hADAR2d, the pDUAL-HFF-eGFP or pDULA-HFF1-dCas13a-hADAR2d plasmid was digested with the NotI restriction enzyme and treated with FastAP thermosensitive alkaline phosphatase (Thermo Scientific) before DNA was recovered with a Gel Extraction Kit (OMEGA). Transformation was carried out with 500 ng of recovered linear DNA. Transformants were selected on Edinburgh minimal medium (EMM) medium supplemented with 50 μg/ml uracil. For episomal expression of Cas13a constructs (either alone or together with crRNA), mCherry-eGFP-W58X constructs (either alone or together with crRNA), the mCherry-eGFP construct (as a positive control) or Tf1 mutant constructs, 100 ng of circular plasmid was used to transform yeast strains and transformants were selected on EMM medium without supplements.

### Measuring growth rates for different *S. pombe* strains

*Schizosaccharomyces pombe* strains were plated in EMM (24) and grown for 4 days. Colonies were picked, employed to seed cultures of 3 ml EMM medium, and grown until mid-log phase. Harvested cells were inoculated in 20 ml of EMM medium with an initial optical density (OD600) of 0.1. The OD600 was measured for each culture at different time points during cell growth.

For comparison of the growth rates on plates, yeast cells were cultured in EMM medium overnight. The cell cultures were then adjusted to 1 OD/ml with EMM medium. The cell suspensions were subsequently diluted to 10^−1^, 10^−2^ or 10^−3^, and 3 μl of each dilution was plated on an EMM or EMM+thiamine plate. Plated cells were allowed to grow for 2 days before being photographed.

### Measuring transcript levels via quantitative PCR

To measure transcript levels in *S. pombe* cells, strains carrying the pDUAL-HFF1-Cas13a, pDUAL-HFF1-Cas13a-tdh1 or pDUAL-HFF1-Cas13a-ade6 plasmid were cultured to late log phase. Yeast cells were then harvested, and total RNA was extracted using the RNeasy Plus Mini Kit (Qiagen), followed by digestion with DNase I to remove possible contamination by genomic DNA. Reverse transcription was performed on total RNA using the RevertAid First Strand cDNA Synthesis Kit (Thermo Scientific) and oligo(dT) primers. Gene transcript levels were quantified via quantitative PCR (qPCR) using the TaKaRa_SYBR^®^*Premix Ex Taq*™ II_RR820Q kit (TAKARA) following the manufacturer’s instructions. Corresponding primer sets and tdh1-79-RT-p5/tdh1-79-RT-p3, ade6-1160-RT-p5/ade6-1160-RT-p3 and act1-1566-RT-p5/Act1-1566-RT-p3 were used for the amplification of *tdh1, ade6* and *act1*, respectively (Supplementary Dataset S1). Note that *act1* was employed as a reference for determining the relative transcript levels of *tdh1* and *ade6*. All qPCR assays were performed in a 20 μl reaction with three technical replicates in 96-well plates, using the StepOnePlus™ Real-Time PCR System (Thermo Fisher Scientific). To normalize RNA inputs, ΔCt values for each sample (e.g. a culture of a strain carrying the plasmid pDUAL-HFF1-Cas13a) were computed by subtracting the Ct values for *act1* from the Ct values for the targets (e.g. *tdh1*). The relative transcript abundance (normalized against *act1*) for each target was obtained as the 2^−ΔCt^ value. The relative transcript abundance of the control sample (the strain carrying the plasmid pDUAL-HFF1-Cas13a) was set as standard ‘1’. The normalized expression of *tdh1* in the strain carrying the plasmid pDUAL-HFF1-Cas13a-rrk1-crRNA-tdh1, or that of *ade6* in the strain carrying the plasmid pDUAL-HFF1-Cas13a-rrk1-crRNA-ade6 was calculated based on comparison against control samples.

### Visualization of red (mCHERRY) and green (eGFP) fluorescence in *S. pombe* cells

*Schizosaccharomyces pombe* strains expressing dCas13a-hADAR2d transfected with different reporter constructs were grown in EMM medium at 30°C to the exponential growth phase. Cells were harvested via centrifugation for 5 min at 1200 *g* at room temperature, then washed twice with 500 μl of distilled water and re-suspended in distilled 60% (v/v) glycerol at 0.5–1.0 × 10^7^ cells/100 μl. A 2.5 μl aliquot of the cell suspension was then placed on a 60 × 24 mm cover slip coated with poly-L-lysine and covered with an 18 × 18 mm cover slip. Fluorescent images were visualized using a ZEISS LSM 880 confocal laser scanning microscope (Axio Imager 2) with a C-Apochromat 40 × /1.2 W KorrM27 objective and a PMT detector. The excitation and emission wavelengths for red fluorescence (mCherry) were 543 and 621 nm, and those for green fluorescence (eGFP) were 488 and 523 nm, respectively.

### Estimation of RNA editing efficiency via flow cytometry

To measure the fluorescence intensities of yeast cells, 20 ml cultures of *S. pombe* strains (with the chromosomal dCas13a-hADAR2d gene) carrying different plasmid constructs (i.e. the positive control (mCherry-eGFP), negative control (mCherry-eGFP-W58X) or mCherry-eGFP-W58X) with various crRNA/pRNA configurations were grown to late log phase. Cells were harvested, and ∼1 × 10^7^ cells were fixed with 4% (v/v) formaldehyde for 2 h. The cells were then washed five times with phosphate-buffered saline (PBS) buffer and resuspended in 1 ml of PBS buffer. Fluorescence intensities at 488 and 561 nm were measured with a BD LSR II SORP flow cytometer (BD Biosciences) with the following configurations: 488 nm blue laser, 505LP LP filter and 525/50 BP filter with the PMT position on E (for eGFP detection); or 561 nm laser, 600 LP filter and 610/20 BP filter with the PMT position on A (for mCHERRY detection). Raw data were analyzed with Flowjo7.5 software (Becton, Dickinson & Company).

To estimate the efficiency of RNA editing based on the ratio of fluorescence intensities between eGFP and mCHERRY (i.e. the ratio of the green fluorescence intensity to the red fluorescence intensity), the ratios of negative control cells and positive control cells were used as the baseline (0%) and maximum (100%), respectively, to normalize and calculate the RNA editing efficiency for samples of different constructs.

### Estimation of RNA editing efficiency via Sanger sequencing

To determine the editing efficiency based on the ratio of edited bases (I) to unedited bases (A), total RNA was extracted using the RNeasy Plus Mini Kit (Qiagen) and then digested with *DNase* I to eliminate possible contamination by genomic DNA. cDNA was synthesized using the RevertAid First Strand cDNA Synthesis Kit (Thermo Scientific) with oligo(dT) primers. DNA fragments of target genes were amplified through PCR using specific primers (Dataset S1), prior to Sanger sequencing (GeneWiz, Suzhou). The RNA editing efficiency for each edited site was estimated based on the ratio of the signal peak height for the edited base to that of the sum of edited and unedited bases, as previously described (25).

### Measuring the transposition frequency of Tf1 mutants

Measurement of Tf1 transposition frequencies was performed as described previously (26,27). Briefly, yeast cells were transformed with mutant Tf1-containing plasmids and allowed to grow on EMM plates for 4 days at 32°C. Colonies were picked and transferred to liquid EMM medium, then cultured for an additional 4 days. The cells were subsequently treated with 5-FOA in liquid EMM containing 50 μg/ml uracil for 2 days, after which 100ul of serial dilutions containing 10^7^, 10^6^, 10^5^ and 10^4^ cells/mL were performed for plating on paired YES plates containing either 5-FOA or both 5-FOA and G418 (26,27). After 4 days, the transposition frequency was estimated by comparing the number of colonies from plates with both G418 and 5-FOA to that from plates with 5-FOA only.

## RESULTS

### Implementation of the type VI CRISPR-Cas13a system in fission yeast

**Figure 1.**
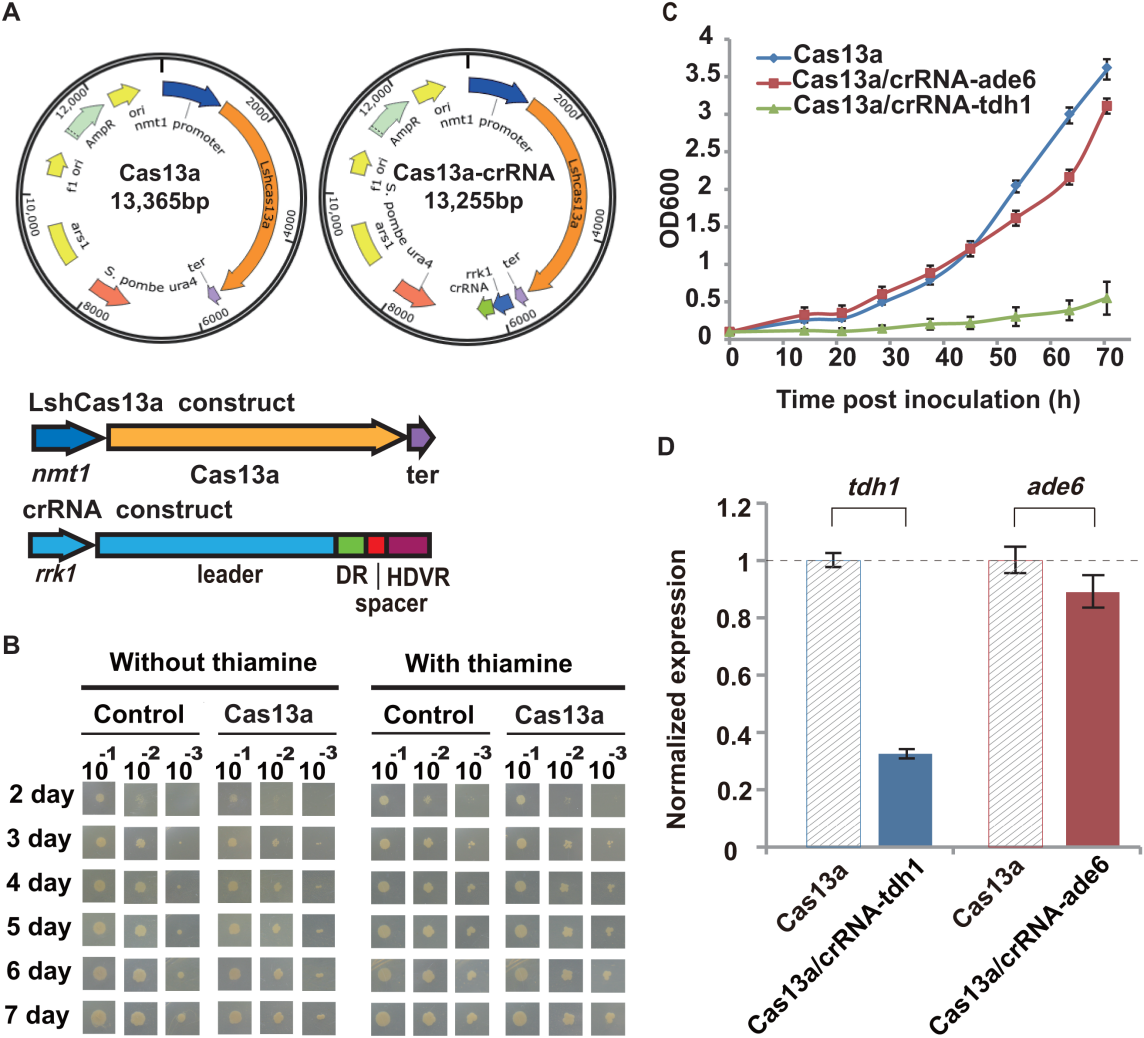
Implementation of the LshCas13a system in fission yeast. (**A**) An *Escherichia coli*–*Schizosaccharomyces pombe* shuttle plasmid (pDUAL-HFF1(29)) was modified to carry either the LshCas13a construct alone or both the LshCas13a and crRNA constructs in the same plasmid. DR, direct repeat region of crRNA; HDVR, hepatitis delta virus ribozyme; *rrk1, rrk1* promoter for generating crRNA (28); ter, *ADH1* terminator. (**B**) Growth phenotype of *S. pombe* strain FY7652, transformed with an empty plasmid or a plasmid encoding only LshCas13a. Control, empty plasmid (pDUAL-HFF1); Cas13a, plasmid encoding LshCas13a. (**C**) Growth curves of *S. pombe* strains expressing either LshCas13a alone, or LshCas13a together with a designed crRNA targeting the *tdh1* (crRNA-tdh1) or *ade6* (crRNA- ade6) transcript. All values are expressed as the mean ± s.e.m. with *n* = 2. (**D**) Knockdown effect of LshCas13a on the *tdh1* or *ade6* gene transcript in *S. pombe*. qPCR was performed on different samples. The normalized expression values of target genes for each sample were determined as described in the ‘Materials and Methods’ section. All values are expressed as the mean ± s.e.m. with *n* = 4, unless otherwise noted. Cas13a: strain carrying the pDUAL-HFF1-Cas13a plasmid; Cas13a/crRNA-tdh1: strain carrying pDUAL-HFF1-Cas13a-rrk1-crRNA-tdh1; Cas13a/crRNA-ade6: strain carrying pDUAL-HFF1-Cas13a-rrk1-crRNA-ade6 (Supplementary Tables S1 and 4).

To implement the type VI CRISPR-Cas13a system in fission yeast, *E. coli*–*S. pombe* shuttle plasmids were constructed to carry the LshCas13a coding sequence either alone (under the control of the *nmt1* promoter) or together with pre-crRNA sequences (under the control of a separate *rrk1* promoter) on the same plasmid (Figure 1A). The *rrk1* promoter was previously employed to generate crRNA for *Streptococcus pyogenes* Cas9 (SpyCas9) in fission yeast (28). Similarly, we included the HDV ribozyme (28) sequence downstream of the pre-crRNA sequences, which helped to remove the 3′-trailing sequences to form clean crRNA. We designed different crRNA constructs to target two endogenous genes, *tdh1* and *ade6*, in *S. pombe*.

The constructed plasmids were transformed into *S. pombe* strains (‘Materials and Methods’ section). The transformed yeast cells were first plated in media containing thiamine, which inhibited the expression of LshCas13a under the *nmt1* promoter. The strains transformed with the empty plasmid (pDUAL-HFF1) (29), or the plasmid containing only the LshCas13a construct were viable and grew at similar rates in the presence of thiamine (Figure 1B). However, when thiamine was withdrawn from the media, we observed slightly slower growth for *S. pombe* strains carrying the plasmid containing the LshCas13a construct than for those carrying the empty plasmid (Figure 1B). Slower growth was often observed when foreign proteins were expressed in yeast (30,31), which suggested possible mild toxicity of the LshCas13a effector toward *S. pombe in vivo*.

We next investigated the knockdown effect of LshCas13a through the introduction of crRNAs targeting the two endogenous gene transcripts (Supplementary Tables S3 and 4). While the presence of crRNA targeting the *tdh1* transcript (crRNA-tdh1) caused significantly slower growth of the yeast cells compared with yeast carrying only LshCas13a, the crRNA targeting *ade6* (crRNA-ade6) showed no significant effect on the cell growth rate (Figure 1C). We further determined the knockdown effect on the *tdh1* and *ade6* transcripts by performing qPCR analysis on total RNA isolated from the transformed yeast strains. We observed a dramatic reduction in the transcript level of the *tdh1* gene, but a less significant reduction of *ade6* gene expression, compared with their expression levels in the controls expressing only LshCas13a (without crRNA) (Figure 1D). It was previously reported that loss of *tdh1* gene led to abnormal mitotic cell cycle and vegetative cell lysis (32,33), which may explain the observed reduction in growth rate in affected yeast cells. Hence, the implemented CRISPR-Cas13a system in fission yeast appeared to knockdown gene transcripts with different efficiencies. The implementation of LshCas13a in *S. pombe* laid the foundation for developing a new toolset for manipulating gene transcripts in this popular model organism.

### Engineering a site-specific RNA editing system using LshCas13a

**Figure 2.**
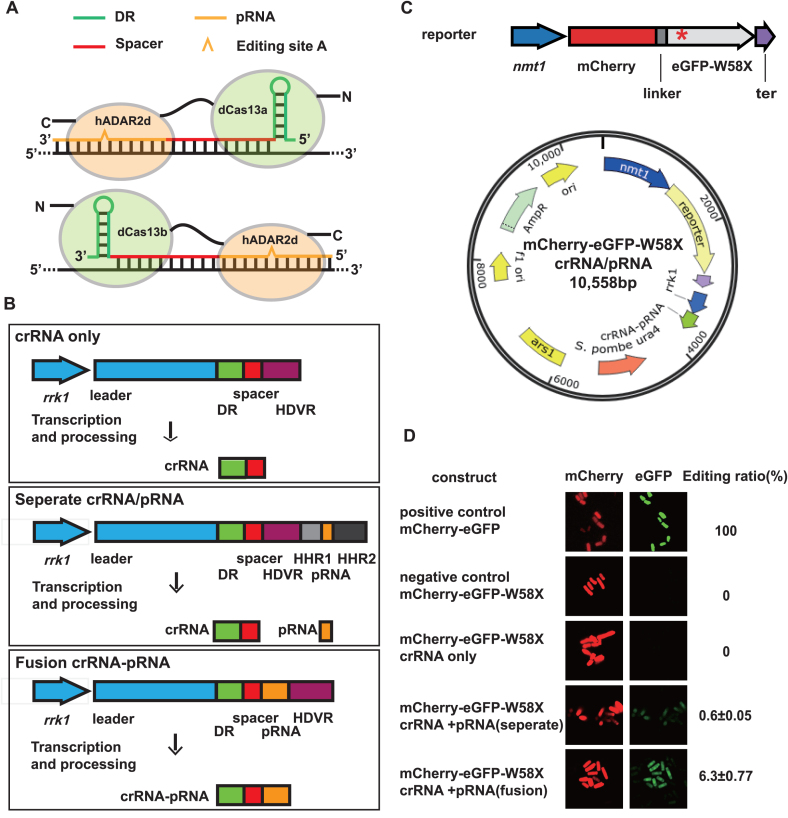
Construction and validation of the dCas13a-mediated system for site-specific editing of mRNA for a fluorescent reporter. (**A**) Schematic comparison between the dCas13a-mediated RNA editing system and that of dCas13b (38). (**B**) Schematic representation of different crRNA/pRNA-expressing constructs in the pDUAL-HFF1 plasmid. The *rrk1* transcription unit was combined with HDVR, HHR1 (52) and HHR2 (28) to generate clean crRNA and pRNA fragments, or fusions of these fragments, as illustrated. DR, direct repeat region of crRNA; HDVR, hepatitis delta virus ribozyme; HHR1, hammerhead ribozyme1; HHR2, hammerhead ribozyme2. (**C**) Schematic representation of the fluorescent reporter construct. The dual-fluorescence reporter (mCherry and eGFP fusion gene) exhibited a stop codon (UAG) at amino acid position 58 of eGFP (named eGFP-W58X), denoted by a red star (*). The reporter construct and the crRNA/pRNA constructs were inserted in the same plasmid, as shown. ter, ADH1 terminator. (**D**) Validation of the dCas13a-mediated site-specific RNA editing of the dual-fluorescent reporter. Confocal images of dCas13a-hADAR2d-expressing *Schizosaccharomyces pombe* cells transfected with different plasmid constructs were obtained using a ZEISS LSM 880 confocal laser scanning microscope (‘Materials and Methods’ section). Red and green images are shown for the same field of cells. Editing ratio was estimated using the ratio of the green fluorescence intensity to the red fluorescence intensity captured via flow cytometry (‘Materials and Methods’ section). All values are expressed as the mean ± s.e.m. with *n* = 2.

The R1278A mutant (dCas13a) of LshCas13a was previously found to have lost target RNA cleavage activity but retained the sequence-specific RNA binding capability of the protein (7). Thus, we explored the possibility of using dCas13a to anchor an RNA deaminase domain to exert a precise RNA editing function in *S. pombe*. As a proof of concept, we designed and engineered a fusion construct by tethering the deaminase domain of human ADAR2 (34,35) (termed hADAR2d) to the C-terminus of dCas13a with a 16-amino acid linker (36) (Figure 2A, upper panel). Although human ADAR2 is known to catalyze the hydrolytic deamination of adenosine (A) to form inosine (I) in double-stranded regions of RNA substrates, its C-terminal domain (hADAR2d) exhibited little activity toward an RNA substrate without an N-terminal dsRNA-binding motif (37). Note that our design using Cas13a had a completely different configuration than that of Cas13b, which presents an opposite structural orientation for RNA binding (Figure 2A, lower panel) (38). To test the dCas13a and hADAR2d fusion construct (designated dCas13a-hADAR2d) *in vivo*, we first generated an *S. pombe* strain that expressed the fusion protein, in which a single copy of the dCas13a-hADAR2d gene under the *nmt1* promoter was integrated into the *leu1* locus in the *S. pombe* chromosome II (Supplementary Figure S3 and ‘Materials and Methods’ section)

To guide the dCas13a-hADAR2d protein to target transcripts for RNA editing, we constructed new plasmids from pDUAL-HFF1 that contained both crRNAs for dCas13a targeting and pairing RNAs (pRNAs) for the formation of the dsRNA substrate required by hADAR2d. The crRNA and pRNA transcription cassette was driven by the *rrk1* promoter (28), and three different constructs were generated: (i) crRNA only, as a control; (ii) crRNA and pRNA as separate molecules; and (iii) crRNA and pRNA as a single fusion molecule (Figure 2B). These plasmid constructs remain episomal after they are transfected into the dCas13a-hADAR2d-expressing *S. pombe* strain.

### dCas13a-mediated site-specific editing of fluorescent reporter mRNA

Our RNA editing system in fission yeast (strain FY7652) consisted of an integrated dCas13a-hADAR2d fusion gene and a modified episomal vector containing crRNA/pRNA constructs. To facilitate the testing of RNA editing activity on target RNA substrates, we next constructed a dual-fluorescence fusion protein reporter consisting of mCherry and eGFP, as described previously (39), but with some modifications (‘Materials and Methods’ section). A single-nucleotide mutation was introduced to generate a stop codon (UAG) at amino acid position 58 of eGFP (designated eGFP-W58X) (Figure 2C, upper panel). Without RNA editing to correct the mutated sequence, the reporter protein emits only red fluorescence. However, once the reporter’s eGFP-W58X mutation is repaired by RNA editing, complete translation is allowed and the protein product emits both red and green fluorescence. The dual-fluorescence fusion protein reporter allowed the efficiency of RNA editing to be estimated simply, by quantifying and comparing the fluorescence intensities at two different channels, thus avoiding the variation of intensity observed using a single-fluorescence protein reporter in different cell samples. To simplify the process, we inserted the reporter construct (mCherry-eGFP-W58X driven by the *nmt1* promoter) in the same plasmids containing different crRNA/pRNA constructs (Figure 2C, lower panel).

While dCas13a-hADAR2d-expressing cells carrying the negative control plasmid (mCherry-eGFP-W58X in pDUAL-HFF1) produced only red fluorescence, cells carrying the positive control (mCherry-eGFP fusion in pDUAL-HFF1) displayed both red and green fluorescence (Figure 2D, first and second rows). When only crRNA was added, only red fluorescence was detected, indicating that no RNA editing had taken place on the reporter RNA (Figure 2D, third row). However, when both crRNA and pRNA were added, both red and green fluorescence were observed in the same cells (Figure 2D, fourth and fifth rows).

The results indicated that the dCas13a-hADAR2d system was capable of editing reporter mRNA at a specific site, and both crRNA and pRNA were required for editing activity. Furthermore, comparing the two different configurations in which crRNA and pRNA were generated, either as two separate RNA molecules or as a single fusion molecule, the fusion construct yielded an apparently stronger green fluorescent signal than that with separate crRNA and pRNA molecules. This finding suggests better efficacy of the fusion molecule in guiding dCas13a-hADAR2d to target RNA or supporting the catalytic reaction of hADAR2d on the dsRNA substrate, or both. The RNA editing efficiency for the crRNA–pRNA fusion construct and for separate crRNA and pRNA molecules was estimated to be ∼6.3% and ∼0.6%, respectively (‘Materials and Methods’ section, Supplementary Dataset S2 and Figure S1).

### Working parameter window for the dCas13a-mediated RNA editing system

While the two-domain dCas13a-hADAR2d protein could be programmed to edit RNA, an important question arose regarding the flexibility of the fusion protein and the size of its working parameter window. As the crRNA and pRNA fusion molecule was found to exhibit a higher efficiency in directing RNA editing by dCas13a-hADAR2d, we proceeded to define its working parameter window using various crRNA–pRNA fusion constructs.

**Figure 3.**
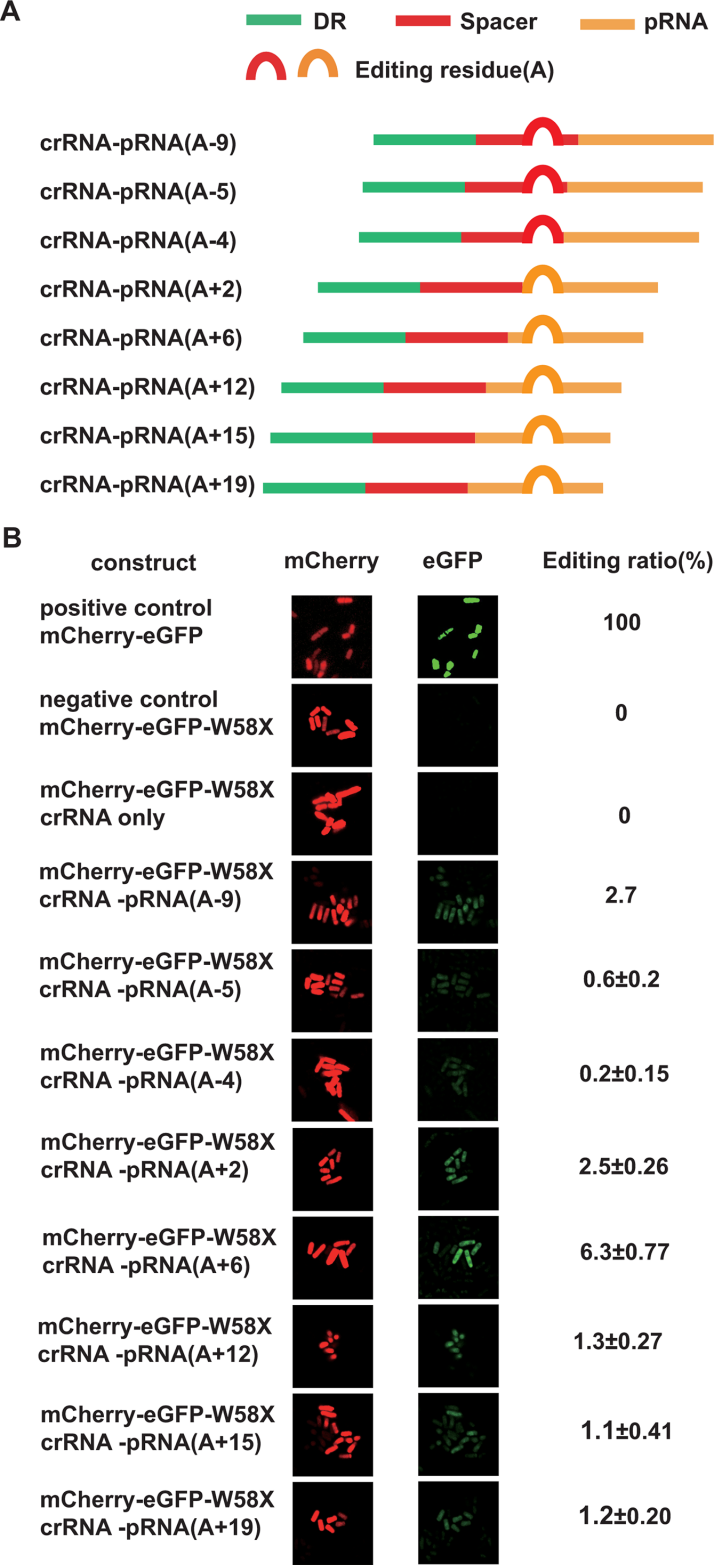
Definition of the working parameter window for dCas13a-mediated RNA editing using a fluorescent reporter. (**A**) Constructs of crRNA–pRNA fusion molecules, as described in Figure 2B. The position of the editing site residue (A) is marked (in brackets). ‘+’ and ‘−’ indicate editing sites inside the pRNA region and crRNA region, respectively, and the numbers indicate the base position from the crRNA/pRNA boundary. (**B**) Visualization of dCas13a-mediated RNA editing activity with the editing site at various positions in crRNA–pRNA fusion molecules. Images were obtained as described in Figure 2D. Editing ratio was estimated using the ratio of the green fluorescence intensity to the red fluorescence intensity captured via flow cytometry (‘Materials and Methods’ section). All values are expressed as the mean ± s.e.m. with *n* = 2.

To map the geometric constrains of the dCas13a-hADAR2d system, we designed and generated a series of crRNA–pRNA fusion constructs by placing the editing site residue (A) at various positions along the crRNA–pRNA fusion sequence (Figure 3A). It should be noted that this was possible due to the flexibility of the 3′ protospacer flanking site for LshCas13a, which allowed an A, U or C residue to be used (7). As a result, we obtained eight different constructs, in which the editing site residues (A) were placed inside either the pRNA region or crRNA region (Figure 3A). The ability of these constructs to direct site-specific RNA editing by dCas13a-hADAR2d was compared by examining the emission of green and red fluorescence in yeast cells carrying each construct. When the editing site residue (A) was positioned inside the crRNA region at −9, −5 or −4 bp, a very weak, but detectable green fluorescence signal was observed; the signal increased as the residue was placed in the pRNA region, peaking around a position +6 bp from the crRNA/pRNA boundary (Figure 3B).

### Length requirement of trans-acting pRNA for efficient RNA editing

**Figure 4.**
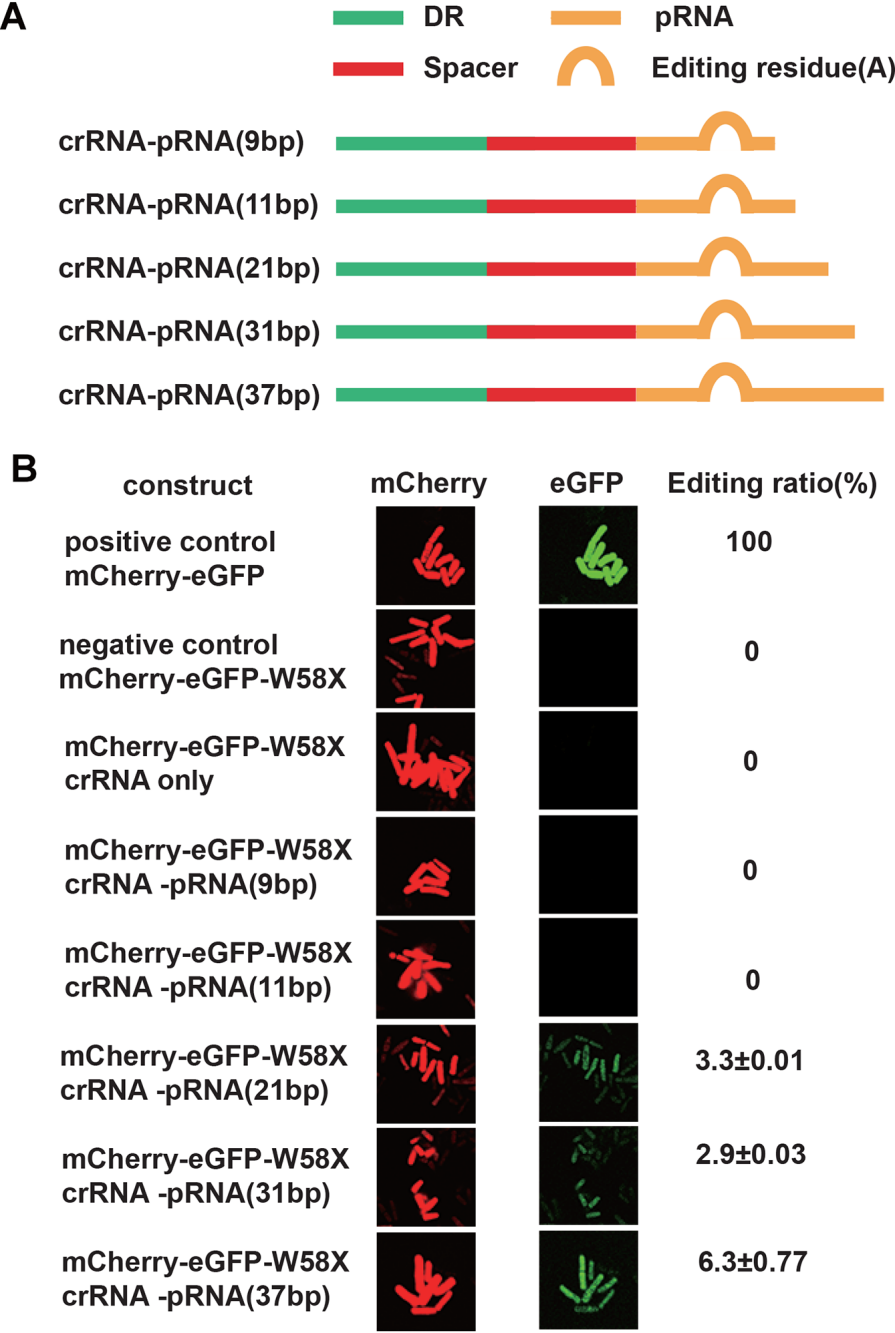
Definition of the length requirement of pRNA for efficient RNA editing by the dCas13a-mediated system. (**A**) Constructs of crRNA–pRNA fusion molecules with variable lengths of pRNA. While the editing site residue (A) was fixed within the pRNA at 6 bp from the crRNA/pRNA boundary, the length of the pRNA varied (9, 11, 21, 31, or 37 bp), as labeled in brackets. (**B**) Visualization of dCas13a-mediated RNA editing activity with various lengths of pRNA. Images were obtained as described in Figure 2D. Editing ratio was estimated using the ratio of the green fluorescence intensity to the red fluorescence intensity captured via flow cytometry (‘Materials and Methods’ section). All values are expressed as the mean ± s.e.m. with *n* = 2.

We next determined the length requirement of pRNA for efficient mRNA editing by dCas13a-hADAR2d in *S. pombe*. Five crRNA–pRNA fusion constructs with varying pRNA lengths of 9, 11, 21, 31 and 37 were generated, with the editing reside (A) fixed within the pRNA region at 6 bp from the crRNA/pRNA boundary (Figure 4A). We observed an increasing green fluorescence signal as the pRNA length was extended, peaking at 37 bp (Figure 4B). Longer pRNAs tended to form more stable dsRNAs with the target RNA, thus facilitating RNA editing by the dCas13a-hADAR2d system in *S. pombe*.

### Application of the dCas13a-mediated RNA editing system to endogenous gene transcripts

To demonstrate the utility of our dCas13a-hADAR2d system in editing gene transcripts other than the fluorescent reporter, we designed and generated crRNA–pRNA constructs targeting randomly selected endogenous genes from the *S. pombe* genome (*tdh1, act1, mel1, ade6, erp5, mug45* and *nmt1*). The expression of these genes ranged from low (e.g. fewer than one mRNA molecule per cell for *mel1* in the single-cell organism vegetative growth phase) to high (e.g. 560 mRNA molecules per cell for *tdh1* during single-cell vegetative growth) (40,41).

The crRNA–pRNA constructs targeting specific residues within the gene transcripts (Table 1) were introduced to the same plasmid construct (under the control of the *rrk1* promoter), as described elsewhere (Figure 2B). The plasmids were then transformed into the *S. pombe* strain with the chromosomal dCas13a-hADAR2d gene. Site-specific RNA editing activity was detected via the sequencing of reverse-transcribed cDNA (‘Materials and Methods’ section). Among the nine target sites, six were found to show detectable RNA editing activity, with the editing efficiency ranging from 7.5 to 54.5% (Table 1 and Supplementary Figure S2). Interestingly, although no editing activity was detected for the *ade6* transcript at position 622A (below detectable levels), such activity was observed at two other sites, 1003A and 1160A, with 7.5 and 9.0% efficiency, respectively. The editing efficiency showed no apparent relationship with either the abundance of transcripts in cells or the G+C content of the sequences flanking the editing sites. Although the *tdh1* and *nmt1* genes displayed comparable transcript abundance (560 and 340 mRNA molecules per cell, respectively) (40), *tdh1* and *nmt1* exhibited dramatically different editing efficiencies, of 54.5 and 0%, respectively. Overall, these results indicated that the dCas13a-hADAR2d system was applicable to a broad range of genes for RNA editing. However, the varying levels of activity observed for different target transcripts suggested the need for further refinement of the system parameters in future applications.

**Table 1.** Editing of endogenous gene transcripts by dCas13a-hADAR2d in S. pombe*

Target gene	Position of target editing residue (A)	^a^ Num of mRNA molecules per cell	^b^Num of G+C/total	^c^ RNA editing efficiency
*tdh1*	79	560	23/65	59%, 50%
*act1*	1566	180	19/65	19%, 12%
*mel1*	921	0.069	26/65	13%, 10%
*ade6*	1160	9.8	27/65	10%, 8%
*ade6*	1003	9.8	24/65	8%, 7%
*ade6*	622	9.8	28/65	Not detected
*erp5*	672	6.8	30/65	14%, 12%
*mug45*	530	0.24	26/65	Not detected
*nmt1*	648	340	25/65	Not detected

*crRNA–pRNA construct was designed for each target with derived optimal settings, i.e. pRNA with a length of 37 bp, and editing site located in pRNA region 6 bp away from crRNA–pRNA boundary. They were placed in the same plasmid (under *rrk1* promoter) as described (Figure 2B).

^a^According to reference (34,35).

^b^Within 65 bp flanking sequence of editing residue.

^c^Detected by Sanger sequencing (‘Materials and Methods’ section and Supplementary Figure S2).

### Restoration of the transposition of retrotransposon Tf1 mutants in fission yeast via dCas13a-mediated RNA editing

**Figure 5.**
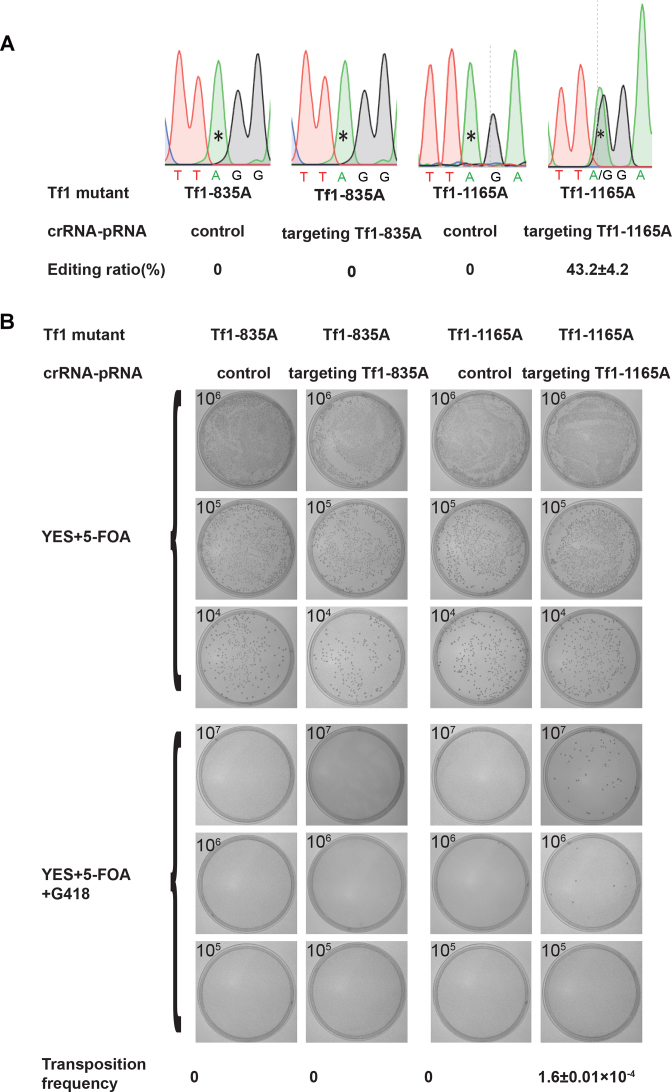
Editing RNA intermediates of retrotransposon Tf1 mutants and restoring transposition with the dCas13a-mediated RNA editing syst*e*m. (**A**) Editing efficiency at residues 835A and 1165A in the Tf1-835A and Tf1-1165A mutants, respectively. Residues targeted by the designed crRNA–pRNA are indicated with *. All values are expressed as the mean ± s.e.m. with *n* = 2. (**B**) Transposition efficiency of Tf1 mutants (as shown in Figure 5A) with mutant residues targeted by the dCas13a-mediated RNA editing system. Cells were replica plated to YES plates with 5-FOA or 5-FOA+G418. All values are expressed as the mean ± s.e.m. with *n* = 3.

Retrotransposon Tf1 undergoes transposition in the genome of fission yeast via an RNA intermediate (26,27). The dCas13a-mediated RNA editing system was used to restore the transposition of retrotransposon Tf1 mutants by manipulating their RNA intermediate via RNA editing (See Supplementary Figure S4 for the design scheme). Two Tf1 mutants were constructed (Tf1-835A and Tf1-1165A), which generated stop codons at positions 99 and 209 of the Tf1 fusion peptide, respectively (Supplementary Figure S4). The two Tf1 mutants could be transcribed under the *nmt1* promoter but failed to retro-transpose, due to a lack of a full-length Tf1 peptide because of the premature stop codons. When the Tf1 mutants were transfected into dCas13a-hADAR2d-expressing cells with the control or the corresponding crRNA–pRNA, Tf1-1165A was edited at residue 1165A with an efficiency of 43.2% (Figure 5A), whereas no editing activity was detected for Tf1-835A at residue 835A, presumably because of the low-efficiency of Tf1-835A targeting crRNA–pRNA. The transposition frequencies of the Tf1 mutants in the presence of the control or the corresponding crRNA–pRNA were measured in plates containing either 5-FOA alone or both 5-FOA and G418 (Figure 5B). While no transposition was detected for Tf1-835A with either the control or Tf1-835A targeting crRNA–pRNA, a transposition frequency of 1.65 × 10^−4^ was observed for Tf1-1165A in the presence of Tf1-1165A targeting crRNA–pRNA (Figure 5B). This utility for retrotransposon manipulation can be expanded and applied to other retrovirus in different hosts, either similar or different to Tf1 in fission yeast. For example, it can manipulate retrovirus sequences at the RNA level, and see how the different variants propagate subsequently. Particularly, by introducing sequence changes before reverse transcription, the system allows interference to take place at the designed stage in the life cycle of a retrovirus, targeting one or multiple genes of choice at once.

## DISCUSSION

We successfully introduced Cas13a (7) from *L. shahii* (LshCas13a) to the model organism *S. pombe*, and used it to target and knockdown endogenous gene transcripts in fission yeast cells. To implement LshCas13a in *S. pombe*, we combined the *rrk1* promoter (transcribed by RNA Pol II) with a ribozyme cassette for *cis*-processing of pre-crRNA. A similar system was previously employed to express the CRISPR-Cas9 system in *S. pombe* (28). To meet the requirements for both the RNA targeting and editing applications of Cas13a, we modified the existing cassette by inserting HDVR and HHR1/HHR2 sequences (Figure 2B). This design proved effective and efficient in generating crRNA alone, or crRNA and pRNA molecules that were either separated or joined. Note that the reported intrinsic RNase activity for the processing of pre-crRNA by Cas13a could be used to simplify our system (8,42). However, in our study design, we were concerned that the inactive LshCas13a (dCas13a) (to be employed in constructing the RNA editing complex) might impair the pre-crRNA processing ability. One area of possible improvement for our system in future work is the testing and utilization of the pre-crRNA processing function of dCas13a, which could greatly reduce the complexity of the RNA editing toolset.

On the basis of the successful implementation of LshCas13a in *S. pombe*, we proceeded to design and engineer a toolset for targeted RNA editing, by tethering inactive Cas13a (dCas13a) to the deaminase domain of human ADAR2 (hADAR2d). The dCas13a-hADAR2d fusion protein was reprogrammed to edit fluorescent reporter mRNA and endogenous gene transcripts. Although no investigation of targeted RNA editing tools for yeast has yet been reported, in mammalian cells, a specifically designed guide-RNA has been introduced to recruit endogenous ADAR2 to edit specific mRNAs (43). This approach is limited to cells expressing the endogenous RNA-editing enzyme ADAR. Additionally, it presents the limitations of background activity from endogenous ADAR and a relatively low binding affinity between the guide-RNA and targets. In other studies, the deaminase domain of human ADAR has been linked to a guide-RNA via either a SNAP-tag strategy or λN-boxB interaction (39,44–46). These methods depend on Watson and Crick base paring for target recognition and binding, in addition to the requirements for the delivery of chemically modified guide-RNA (for SNAP-tag) to living cells (46–48). Our dCas13a-hADAR2d system provides an attractive alternative for RNA editing applications. Cas13a-crRNA and target-RNA binding is highly specific and selective. dCas13a (R1278A mutated LshCas3a) exhibits an even stronger binding affinity toward its target RNA (*K*_D_ ∼ 7 nM) than wild-type LshCas13a (*K*_D_ ∼ 46 nM) (7). This high binding affinity makes Cas13a suitable for a range of applications, such as tagging for molecule localization and subcellular trafficking, RNA modification, and enrichment of specific RNA transcripts with their partners (6,9,12).

While our data demonstrated the flexibility of dCas13a-hADAR2d at the editing residue position, we were surprised to observe detectable, albeit very low activity of RNA editing for sites inside the spacer region of crRNA (Figure 3). The reported crystal structure of LshCas13a in complex with crRNA suggests that nucleotide bases 24–28 of the crRNA are exposed on the outside of the LshCas13a structure (42). Furthermore, based on the crystal structure of another closely related Cas13a from *Lachnospiraceae bacterium* (LbaCas13a), the first 13 nucleotides (C1–G13) of the spacer in the LbaCas13a/crRNA complex are well ordered and sequestered within the LbaCas13a protein, whereas the rest 11 (G14–C24) were disordered and exposed in solvent (9). The disordered regions may suggest a possible opening for access by hADAR2d, resulting in residue RNA editing activity in the spacer region.

The utilization of a different member of the Cas13 family, Cas13b from *Prevotella sp. P5-125* (PspCas13b), to mediate RNA editing in human cell lines has also been reported (38). With an improved hyperactive hADAR2d variant (E488Q), the efficiency of editing using the PspCas13b/hADAR2d(E488Q) system is comparable to or better than that of the guide-RNA approaches discussed above (43,46–48). The performance of our dCas13a-hADAR2d system in *S. pombe* varied for different targets, with an RNA editing efficiency of up to 54.5% being achieved for *tdh1* (Table 1). There are large differences in the environment, design and configurations of these two systems. Notably, Cas13a-crRNA and Cas13b-crRNA complexes bind to their target RNAs in opposite orientations (10,38). Thus, our dCas13a-mediated RNA editing system has a completely different configuration from the dCas13b-mediated system (38) (Figure 2A). In addition, factors such as gene transcript abundance, RNA secondary structure and the molar ratio of dCas13a-hADAR2d to either crRNA–pRNA or target transcripts contribute to the variation in editing efficiency. For example, an excessive amount of crRNA–pRNA over dCas13a-hADAR2d molecules would prevent the dCas13a-hADAR2d/crRNA–pRNA complex from acting on target RNAs by pre-occupying their proto-spacer sites on target RNA. It was also found that the formation of a Cas13a and crRNA complex was a pre-requisite for their recognition and binding of target RNA (9,42). Further investigation is warranted to illustrate the mechanism underlying the specificity and efficiency of the Cas13-mediated RNA interference system. The dCas13b-mediated RNA editing system generated a number of off-target editing events, which were driven by hADAR2d editing activities independent of dCas13b targeting (38). Further, it was shown that mutated hADAR2d, e.g., E448Q/T375G mutants that destabilized hADAR2d-RNA binding, decreased the off-target editing activities. While RNA editing by ADARs was widely found in insects and vertebrates (49,50), there is no native ADAR enzyme in yeast *S. pombe*, and presumably no natural substrates (that have to form appropriate dsRNA structure) exist. So it is likely that very few, if any, off-target editing sites exist in *S. pombe*. Supporting our speculation, in yeast *S. cerevisiae* only around a dozen editing sites were found in mRNA substrates when transfected with a full-length human ADAR2 vector (51).

While we have shown that Cas13a can be repurposed and implemented for RNA interference and RNA editing tasks in *S. pombe*, we foresee the potential improvement and expansion of this system for many other functions, to address fundamental questions regarding the modulation of gene functions at the RNA level using the model system of fission yeast. To improve the efficiency of the CRISPR-Cas13-related tool, it can be enhanced with new functionalities. For example, our dCas13a-hADAR2d system in *S. pombe* can be further improved by simply using the E488Q variant of hADAR2d. Additionally, we may consider expanding its RNA editing activity by replacing hADAR2d with an RNA-editing enzyme with a different specificity, such as a member of the APOBEC family of enzymes that catalyze cytidine to uridine conversion. The available molecular tools for manipulating RNA sequences are limited, and this RNA manipulation platform is especially valuable for perturbing model organisms such as *S. pombe*. Our implementation of the CRISPR-13a system in fission yeast serves as a stepping stone toward a much-promised new family of toolsets that target gene transcripts for a wide range of research and biotechnological applications.

## Supplementary Material

Supplementary Data
