# Supplementary material for: Implementation of the CRISPR-Cas13a system in fission yeast and its repurposing for precise RNA editing
Source: Nucleic Acids Res. 2018 May 31;46(15):e90. doi: 10.1093/nar/gky433 (PMC6125684; doi:10.1093/nar/gky433)
Supplement: Supplementary Data [file gky433_supplemental_files.zip › Supplementary Information 1_v4.9.docx]

**Supporting Information**

**Implementation of CRISPR-Cas13a system in fission yeast and its repurposing for precise RNA editing**

Xinyun Jing^1,#^, Bingran Xie^1,#^, Longxian Chen^1^, Niubing Zhang^1,3^, Yiyi Jiang^2^ , Hang Qin^1^, Hongbing Wang^4^, Pei Hao^2,^*, Sheng Yang^1,^* and Xuan Li^1,^*

**SUPPLEMENTARY METHODS**

**Construction of Cas13a and dCas13a expression vectors**

Plasmids pDUAL-HFF1-Cas13a, pDUAL-HFF1-dCas13a and pDUAL-HFF1-Cas13a-hADAR2d were generated by inserting the PCR fragments of LshCas13a, dCas13a, and dCas13a-hADAR2d into the NdeI/NcoI restriction sites of pDUAL-HFF1. The Cas13a gene fragment was amplified using plasmid pC001 (Table S1) as template and primers Cas13a-P5 /Cas13a-P3. The dCas13a gene fragment was obtained by overlap PCR using plasmid pC001 as template and primers Cas13a-P5, Cas13a-mut-mid-P3, Cas13a-mut-mid-P5 and Cas13a-P3. The dCas13a-hADAR2d gene fragment was obtained by overlap PCR using dCas13a gene fragment and ADAR2 gene fragment as template and primers Cas13a-P5, XTEN-dCas13a-P3, XTEN-hADAR2d-P5, hADAR2d-P3.

**Construction of dual-fluorescence reporter vectors**

To generate plasmids pDUAL-HFF1-eGFP, pDUAL-HFF1-mCherry-eGFP and pDUAL-HFF1-mCherry-eGFP-W58X, fragments of eGFP, mCherry-eGFP and mCherry-eGFP-W58X were obtained with from PCR amplification or digestion of intermediate cloning plasmids pKS-mCherry-eGFP or pKS-mCherry-eGFP-W58X (Table S2), and were cloned into the NheI/BglII sites in plasmid pDUAL-HFF1. To produce the intermediate cloning plasmids pKS-mCherry-eGFP and pKS-mCherry-eGFP-W58X, we first amplified the mCherry-linker fragment by overlap PCR using plasmid pmCherry Paxillin (Addgene: 50526) and plamid pLinker (synthesized by Genwiz, Suzhou, China) as templates using primers mCherry-P5, mCherry-linker-P3, mCherry-linker-P5 and linker-P3. The mCherry-linker gene fragment was cloned into the KpnI/XbaI sites of plasmid pBluescript II KS(+) (simplified as pKS in Table S1) producing intermediate plasmid pKS-mCherry-linker. Second, we amplified eGFP gene fragment using plasmid pEGFP-N1-FLAG (Addgene: 60360) as template with primers eGFP-P5/ eGFP-P3. Meanwhile we amplified the eGFP-W58X gene fragment by overlap PCR using plasmid pEGFP-N1-FLAG as template with primers eGFP-P5, eEGFP-mut-P3, eEGFP-mut-P5, eGFP-P3. Third, the eGFP and eGFP-W58X gene fragment were cloned into XbaI/SacI sites of plasmid pKS-mCherry-linker generating plasmids of pKS-mCherry-eGFP and pKS-mCherry-eGFP-W58X.

**Construction of crRNA expression vectors**

The intermediated crRNA cloning construct was built by synthesizing the gene composing *rrk1* promoter, leader RNA, BspQI placeholder, HDVR Ribozyme, BsaI placeholder and Hammerhead Ribozyme (synthesized by Genwiz, Suzhou, China), and digesting the synthesized DNA with ClaI/EcoRV restriction enzyme, before cloning them into the pKS plasmid (BspQI and BsaI restriction enzyme recognition sites disappeared by mutation) yielding pKS-crRNA-backbone vector. Complementary primers used for generation of crRNA and pRNA were combined (5 μl of 100 μM solution) in Tris buffer and annealed by heating to 95 °C for 5 min, followed by a gradual cooling to 45 °C at a rate of 0.1 °C per second to generate dsDNA substrates with sticky end. crRNA were cloned into BspQI placeholder of plasmid pKS-gRNA-backbone, producing intermediate plasmids in Table S2. pRNA was cloned into the *Bsa*I placeholder of plasmid pKS-rrk1-crRNA-control yielding plasmid pKS-rrk1-crRNA-pRNA-separate as Table S2.

The crRNA expressing plasmids was constructed using ClonExpress ® II One Step Cloning Kit. The crRNA fragments were amplified using the intermediated crRNA cloning construct (as in Table S2) as templates with primers pDUAL-SpeI-T3 and pDUAL-XhoI-T7. The plasmids pDUAL-HFF-mCherry-eGFP-W58X, pDUAL-HFF1-Cas13a, and pDUAL-HFF1 were digested with SpeI and PspXI. The pDUAL-HFF1-Cas13a SpeI/PspXI fragment and the crRNA-ade6-1160, crRNA-tdh1-79 PCR fragments were recombined using ClonExpress ® II One Step Cloning Kit generating plasmids as in Table S4. pDUAL-HFF-mCherry-eGFP-W58X SpeI/PspXI fragment and crRNA-28bp, crRNA-37bp, crRNA-39BP, crRNA-49bp, crRNA-59bp, crRNA-65bp, crRNA-(-9)-65, crRNA-(-5)-65, crRNA-(-4)-65, crRNA-2-65, crRNA-12-65, crRNA-15-65, crRNA-19-65 PCR fragments were recombined using ClonExpress ® II One Step Cloning Kit, generating plasmids as in Table S5. pDUAL-HFF-mCherry-eGFP-W58X SpeI/PspXI fragment and crRNA-act1-1566, crRNA-ade6-622, crRNA-ade6-1002, crRNA-ade6-1160, crRNA-erp5-672, crRNA-mel1-921, crRNA-mug45, crRNA-nmt1-648, crRNA-tdh1-79 PCR fragments were recombined using ClonExpress ® II One Step Cloning Kit generating plasmids as in Table S6.

**Construction of retrotransposon Tf1 mutants**

The plasmid pHL414-Tf1-835A and pHL414-Tf1-1165A were constructed by recombining pHL414 digested with XhoI enzyme with the Tf1-835A and Tf1-1165A mutant fragment. The Tf1-835A mutant fragments were amplified by overlap PCR using primers TF1-XhoI-nmt1-P5/ Tf1-835-mutant-P3 and TF1-835-mutant-P5/ neo-P3. The Tf1-1165A mutant fragments were produced in the same way as Tf1-835A mutant using primers TF1-XhoI-nmt1-P5/Tf1-1165-mutant-P3 and TF1-1165-mutant-P5/neo-P3. The plasmids with mutant Tf1 and crRNA-pRNA expressing fragment were built by inserting the crRNA-pRNA expressing fragments into the NheI site of the plasmids pHL414-Tf1-835A or pHL414-Tf1-1165A. The crRNA-pRNA expressing fragments were amplified from using the intermediated crRNA cloning construct with primers T7/T3-XbaI and then digested with XbaI enzyme.

**SUPPLEMENTARY FIGURES**

**Figure S1**


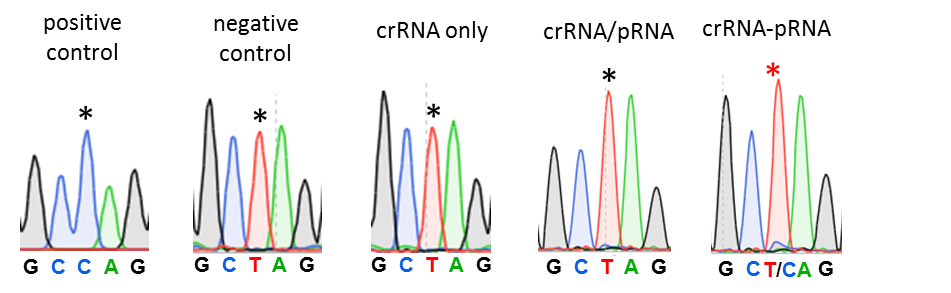


**Figure S1.** Electropherograms from RT-PCR products of *S. pombe* with chromosomal dCas13a-ADAR2d gene carrying different plasmids construct (*SI Methods* and Table S5). Positive control, mCherry-eGFP in pDUAL-HFF1; negative control, mCherry-eGFP-W58X in pDUAL-HFF1.

**Figure S2**


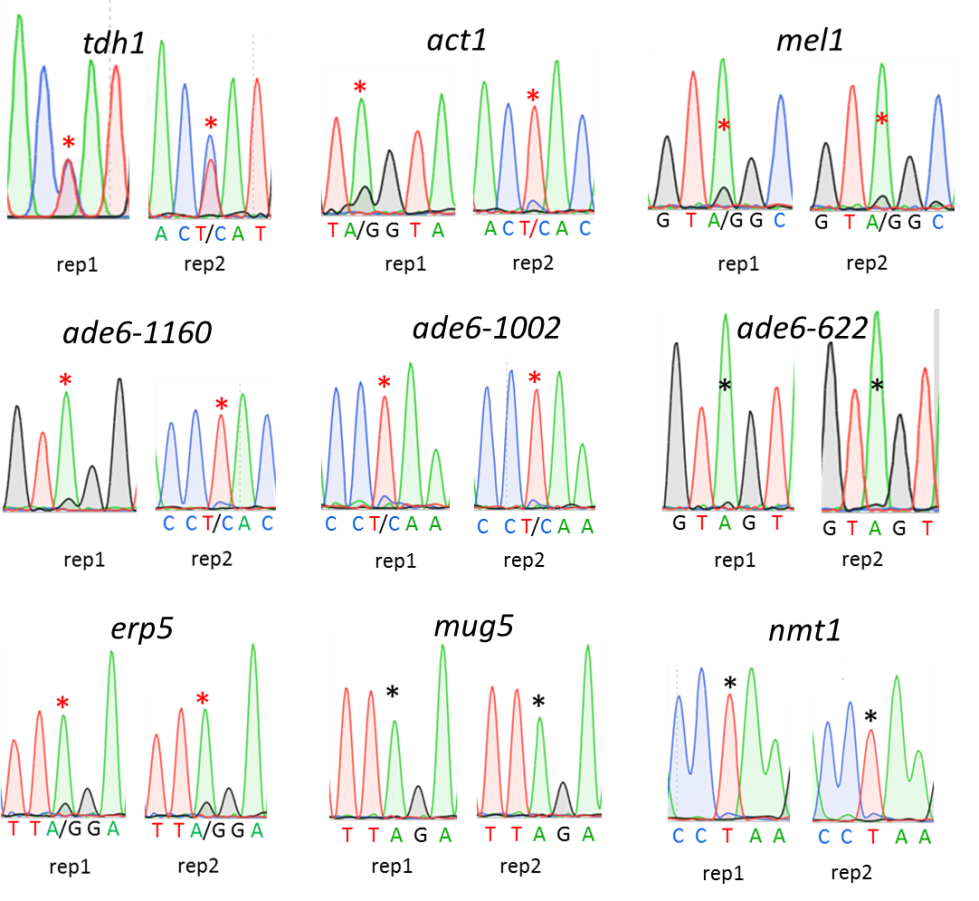


**Figure S2.** Electropherograms from RT-PCR products of *S. pombe* carrying plasmids with episomal dCas13a-ADAR2d gene and different crRNA-pRNA construct targeting endogenous gene transcripts (*SI Methods* and Table S5). crRNA-pRNA construct was designed for each target with derived optimal settings, i.e. pRNA with a length of 37 bp, and editing site located in pRNA region 6 bp away from crRNA-pRNA boundary. They were placed in the same plasmid (under the control of *rrk1* promoter) as described (Fig. 2*B*)

**Figure S3**

**Figure S3.** Scheme for chromosomal expression of dCas13a-hADAR2d in *S. pombe*. dCas13a-hADAR2d fusion gene (under the control of *nmt1* promoter) was integrated into the leu1 locus in *S. pombe* chromosome II (Materials and Methods). Ter, ADH1 terminator.

**Figure S4**

**
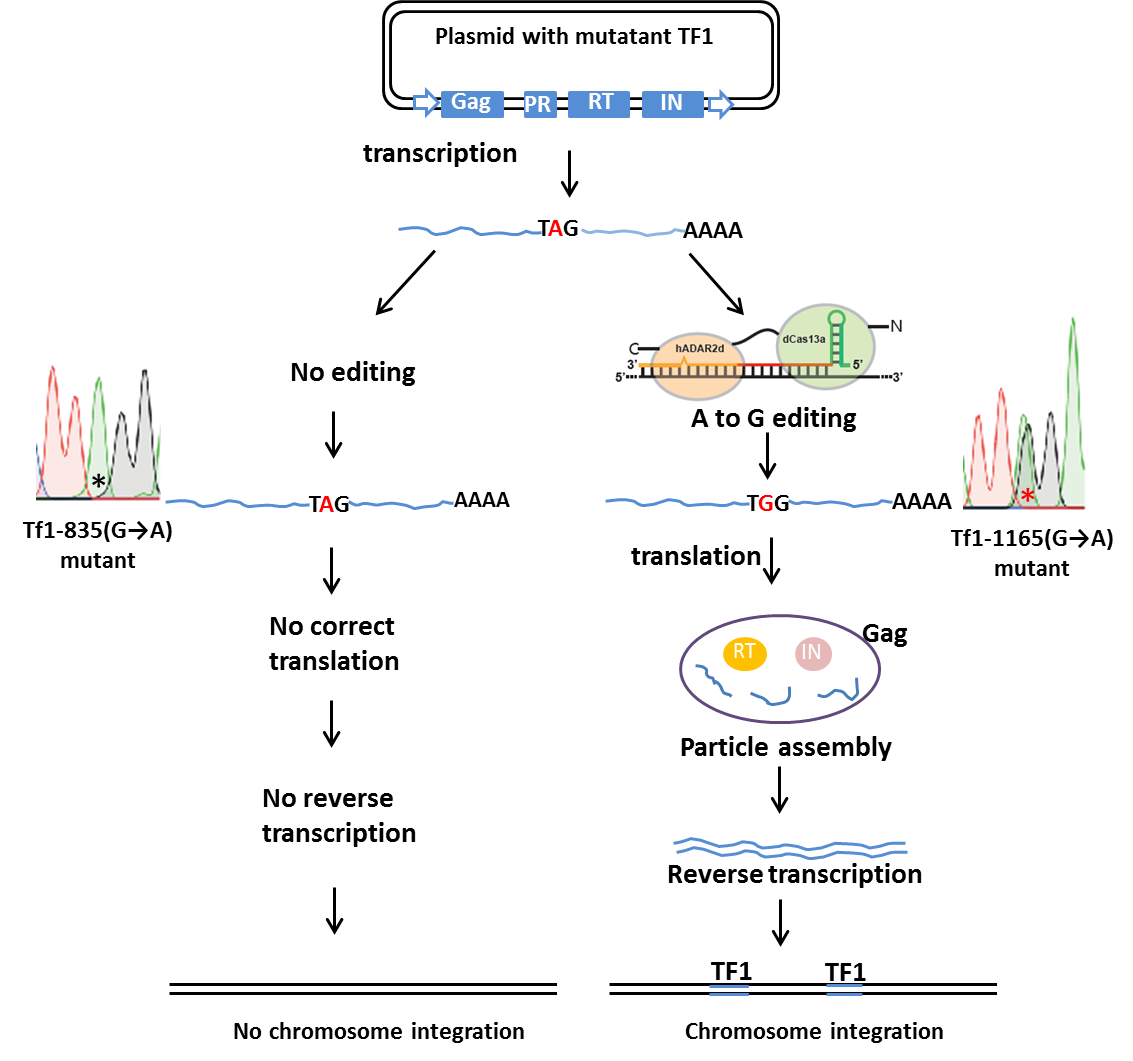
**

**Figure S4.** Site directed A to I editing restored mutant Tf1transposition activity in *S. pombe*. Full-length Tf1 is transcribed in to mRNA. If the Tf1 nonsense mutation like 1165(G→A) in mRNA is repaired by the dCas13a-hADAR2d dependent RNA editing system (right), the Tf1 mRNA can be translated into a single primary product, and then processed into Gag, protease(PR), reverse transcriptase(RT) and integrase (IN). The RT reverse-transcribes the mRNA into cDNA that associates with IN. Once transported into the nucleus, the IN integrates the cDNA at a new position in the genome. If the Tf1 nonsense mutation as 835(G→A) was not repaired by the dCas13a-hADAR2d dependent RNA editing system (left), the translation stops in 99 codon, which resulted in no reverse-transcription and no transposition.

**Figure S5**

atggggaacctgttcggacacaagcggtggtacgaggtgcgagataagaaagacttcaagatcaagcgaaaggtgaaagtcaagcggaattatgacgggaacaagtacattctgaacatcaacgaaaacaacaacaaagagaagatcgacaacaacaagttcatcagaaagtacatcaactacaagaaaaacgataatattctgaaggagttcactaggaaatttcatgcaggaaatatcctgttcaaactgaagggcaaagaagggatcattagaattgagaataacgacgacttcctggagacagaggaagtggtgctgtatatcgaggcctacggcaagagcgagaagctgaaagcactggggatcacaaagaaaaagatcattgacgaggccatcaggcagggcattactaaggacgataaaaagatcgagatcaagcgacaggagaacgaggaagagatcgaaattgacatccgggatgagtacactaataagaccctgaacgactgctccatcattctgcgcatcattgaaaacgatgaactggagacaaagaagagcatctacgagatcttcaagaacatcaatatgagcctgtataagatcatcgagaagatcatcgaaaacgagacagaaaaggtgtttgaaaatagatactacgaagagcacctgagggagaagctgctgaaagacgataagattgacgtgatcctgaccaacttcatggaaatccgggagaagatcaagtctaatctggagatcctgggcttcgtgaagttttacctgaacgtcggcggggacaaaaagaaaagtaaaaataagaaaatgctggtggaaaagattctgaacatcaatgtggatctgaccgtcgaggacattgccgatttcgtgatcaaggagctggaattttggaacatcacaaagcgcattgagaaagtgaagaaagtcaataacgagttcctggagaagcggagaaatcggacatacatcaagtcctatgtgctgctggacaagcacgaaaagtttaaaatcgagagagaaaacaagaaggataagatcgtgaagttctttgtcgagaacattaagaacaactctatcaaggaaaagattgagaagatcctggctgagttcaagatcgacgagctgattaagaaactggagaaggaactgaagaaagggaactgtgataccgagatcttcggaatctttaagaagcattacaaggtgaacttcgacagcaagaaattttccaagaaatctgatgaagagaaggagctgtataagatcatctacagatacctgaagggcagaattgaaaaaatcctggtgaacgagcagaaggtcagactgaagaaaatggagaagatcgagatcgaaaagattctgaatgaaagtatcctgtcagagaaaattctgaagagagtgaaacagtatacactggagcacattatgtacctggggaagctgaggcataacgacatcgatatgaccacagtgaatactgacgatttcagccgcctgcacgccaaggaagagctggacctggaactgatcaccttctttgccagcacaaatatggagctgaacaagatcttttcccgagaaaacatcaacaacgacgagaacatcgatttctttggaggcgaccgggagaagaactatgtgctggataagaaaatcctgaatagtaagatcaagatcatccgcgacctggatttcatcgataacaagaacaacatcacaaacaacttcattcgaaagtttacaaagatcggcactaatgaaaggaaccgcatcctgcatgccatttccaaagagagggacctgcaggggactcaggacgattacaacaaagtgatcaacatcattcagaatctgaagatctccgatgaagaggtgagcaaagctctgaacctggacgtggtctttaaggacaagaaaaacatcatcacaaagatcaatgacatcaagatctctgaagagaacaacaacgatatcaagtatctgcccagcttcagcaaagtgctgcccgaaatcctgaacctgtaccgcaacaatcccaagaatgagccttttgacacaatcgagactgaaaaaattgtgctgaacgctctgatctacgtcaataaggagctgtataagaaactgatcctggaggacgatctggaagagaacgagtccaagaatatcttcctgcaggaactgaagaaaaccctgggcaacattgacgaaatcgatgagaacatcatcgagaactactacaagaacgcacagatttctgccagtaaggggaacaacaaggcaatcaagaaatatcagaagaaagtgatcgagtgctacattggatatctgcgcaaaaactacgaagagctgttcgacttttcagacttcaagatgaacatccaggaaatcaagaaacagattaaggacatcaacgataacaagacttatgagcggatcaccgtgaaaaccagcgacaaaaccattgtcatcaacgacgatttcgagtacatcatttctatctttgcactgctgaacagtaatgccgtgattaataagatccgaaacagattcttcgccaccagcgtgtggctgaacacctcagaataccagaatatcattgacatcctggatgagattatgcagctgaataccctgcggaacgaatgcatcacagagaactggaatctgaacctggaagagttcattcagaagatgaaagagatcgaaaaggatttcgacgacttcaagatccagactaagaaagaaatcttcaacaactactacgaggacatcaagaacaacattctgaccgagtttaaagacgatatcaacggctgtgatgtgctggaaaagaaactggagaagattgtcatcttcgacgatgaaaccaagttcgagatcgacaagaaatccaacatcctgcaggatgaacagagaaagctgtctaacatcaacaagaaggacctgaagaaaaaggtggatcagtatatcaaggacaaagatcaggagatcaagtctaaaatcctgtgcaggatcattttcaacagtgactttctgaaaaagtacaagaaggaaatcgacaatctgattgaggatatggagtctgaaaatgagaacaagttccaggagatctactatcccaaggaacggaagaacgagctgtatatctacaaaaagaatctgttcctgaacatcggaaatcctaactttgacaagatctacggcctgattagcaacgacatcaagatggccgatgctaaattcctgtttaatatcgatggaaagaacatcagaaagaacaaaatcagtgagatcgacgctattctgaagaatctgaacgataaactgaacggctactcaaaggaatacaaggagaagtacatcaaaaagctgaaggagaacgacgatttctttgcaaagaacatccagaataagaactacaaatccttcgaaaaggactataaccgcgtgtctgagtacaaaaagattcgagatctggtcgagttcaactatctgaacaaaatcgagtcctacctgattgacatcaactggaagctggctattcagatggcaagattcgaaagggatatgcactatatcgtgaatggactgagggagctgggcatcattaagctgtcaggctataacaccgggatcagcagggcatacccaaagcgcaatggaagcgacggcttttacactaccacagcctactacaagttctttgatgaagagtcctacaagaagttcgagaagatttgctacgggtttggaatcgacctgagcgaaaattccgagatcaacaagcctgaaaatgagagcattgctaactatatctcccatttctacatcgtgagaaatccatttgccgactacagtattgctgagcagatcgatcgggtgagcaacctgctgtcatatagcacacgctacaacaattcaacttatgccagcgtgttcgaagtctttaaaaaggacgtgaatctggactacgatgagctgaaaaagaaattcaaactgatcggcaacaatgatattctggagcgcctgatgaagcccaagaaagtgagcgtcctggaactggagtcctacaacagtgactacattaagaatctgatcattgaactgctgaccaaaatcgagaatactaacgataccctgaaatctggttctgaaactcctggtacttctgaatctgctactcctgaattgcacttggatcagacgccatctcgccagcctattcccagtgagggtcttcagctgcatttaccgcaggttttagctgacgctgtctcacgcctggtcctgggtaagtttggtgacctgaccgacaacttctcctcccctcacgctcgcagaaaagtgctggctggagtcgtcatgacaacaggcacagatgttaaagatgccaaggtgataagtgtttctacaggaacaaaatgtattaatggtgaatacatgagtgatcgtggccttgcattaaatgactgccatgcagaaataatatctcggagatccttgctcagatttctttatacacaacttgagctttacttaaataacaaagatgatcaaaaaagatccatctttcagaaatcagagcgaggggggtttaggctgaaggagaatgtccagtttcatctgtacatcagcacctctccctgtggagatgccagaatcttctcaccacatgagccaatcctggaagaaccagcagatagacacccaaatcgtaaagcaagaggacagctacggaccaaaatagagtctggtgaggggacgattccagtgcgctccaatgcgagcatccaaacgtgggacggggtgctgcaaggggagcggctgctcaccatgtcctgcagtgacaagattgcacgctggaacgtggtgggcatccagggatccctgctcagcattttcgtggagcccatttacttctcgagcatcatcctgggcagcctttaccacggggaccacctttccagggccatgtaccagcggatctccaacatagaggacctgccacctctctacaccctcaacaagcctttgctcagtggcatcagcaatgcagaagcacggcagccagggaaggcccccaacttcagtgtcaactggacggtaggcgactccgctattgaggtcatcaacgccacgactgggaaggatgagctgggccgcgcgtcccgcctgtgtaagcacgcgttgtactgtcgctggatgcgtgtgcacggcaaggttccctcccacttactacgctccaagattaccaagcccaacgtgtaccatgagtccaagctggcggcaaaggagtaccaggccgccaaggcgcgtctgttcacagccttcatcaaggcggggctgggggcctgggtggagaagcccaccgagcaggaccagttctcactcacgccctga

**Figure S5.** Gene sequences of dCas13a-hADAR2d in the context of pDUAL-HFF1 plasmid, under the promoter of *nmt1* and ADH1 terminator. Nucleotides for R1278A mutant of Cas13a protein, green background; XTEN linker sequence(1) with codon optimized for expression in *S. pombe*: magenta background.

**Figure S6**

ATGGTGAGCAAGGGCGAGGAGGATAACATGGCCATCATCAAGGAGTTCATGCGCTTCAAGGTGCACATGGAGGGCTCCGTGAACGGCCACGAGTTCGAGATCGAGGGCGAGGGCGAGGGCCGCCCCTACGAGGGCACCCAGACCGCCAAGCTGAAGGTGACCAAGGGTGGCCCCCTGCCCTTCGCCTGGGACATCCTGTCCCCTCAGTTCATGTACGGCTCCAAGGCCTACGTGAAGCACCCCGCCGACATCCCCGACTACTTGAAGCTGTCCTTCCCCGAGGGCTTCAAGTGGGAGCGCGTGATGAACTTCGAGGACGGCGGCGTGGTGACCGTGACCCAGGACTCCTCCCTGCAGGACGGCGAGTTCATCTACAAGGTGAAGCTGCGCGGCACCAACTTCCCCTCCGACGGCCCCGTAATGCAGAAGAAGACCATGGGCTGGGAGGCCTCCTCCGAGCGGATGTACCCCGAGGACGGCGCCCTGAAGGGCGAGATCAAGCAGAGGCTGAAGCTGAAGGACGGCGGCCACTACGACGCTGAGGTCAAGACCACCTACAAGGCCAAGAAGCCCGTGCAGCTGCCCGGCGCCTACAACGTCAACATCAAGTTGGACATCACCTCCCACAACGAGGACTACACCATCGTGGAACAGTACGAACGCGCCGAGGGCCGCCACTCCACCGGCGGCATGGACGAGCTGTACAAGGAATTCtggaacatggcctcccgaggagcccagacagctgcagccacagctccccgtatcaagaaatttgccatctatcgatgggacccagacaaggctggagacaaacctcatTCTAGAGTGAGCAAGGGCGAGGAGCTGTTCACCGGGGTGGTGCCCATCCTGGTCGAGCTGGACGGCGACGTAAACGGCCACAAGTTCAGCGTGTCCGGCGAGGGCGAGGGCGATGCCACCTACGGCAAGCTGACCCTGAAGTTCATCTGCACCACCGGCAAGCTGCCCGTGCCCTGGCCCACCCTCGTGACCACCCTGACCTACGGCGTGCAGTGCTTCAGCCGCTACCCCGACCACATGAAGCAGCACGACTTCTTCAAGTCCGCCATGCCCGAAGGCTACGTCCAGGAGCGCACCATCTTCTTCAAGGACGACGGCAACTACAAGACCCGCGCCGAGGTGAAGTTCGAGGGCGACACCCTGGTGAACCGCATCGAGCTGAAGGGCATCGACTTCAAGGAGGACGGCAACATCCTGGGGCACAAGCTGGAGTACAACTACAACAGCCACAACGTCTATATCATGGCCGACAAGCAGAAGAACGGCATCAAGGTGAACTTCAAGATCCGCCACAACATCGAGGACGGCAGCGTGCAGCTCGCCGACCACTACCAGCAGAACACCCCCATCGGCGACGGCCCCGTGCTGCTGCCCGACAACCACTACCTGAGCACCCAGTCCGCCCTGAGCAAAGACCCCAACGAGAAGCGCGATCACATGGTCCTGCTGGAGTTCGTGACCGCCGCCGGGATCACTCTCGGCATGGACGAGCTGTACAAGTCCGGATAA

**Figure S6.** Gene sequences of mCherry-eGFP-W58X in the context of pDUAL-HFF1 plasmid, under the promoter of *nmt1* and ADH1 terminator. Nucleotide for the W58X mutant of eGFP protein, red background.

**Figure S7**

TTTTGCTTATGTTGGTGGTAGTTGGCATGCGTAGACTGATGACTAGTCAGCAAGGAGCGTAGAACAGTCACACTCGTTATATATGTGCTTCCAAGAAAACTCAAGAATTTACCATTAGCAAACACTTTTTTGAAATGTTAGACATTTAAATGACGAAGGCATATAGAAGCTTTGAATAGGTGTTGTAAAGTGTTGATTTATGTGACGCTGAGGGTGCGCATGAAAGGAATGTTGGGTCACGATTATTAAACAGTTTGCTAGCTTGGACACTTGAGTATTGGAAGTTGTTGAATTCTAAAAAACTTTCAGTTGATTTGAATAGTTGCTGTTGCCAAAAAACATAACCTGTACCGAAGAAccaccccaatatcgaaggggactaaaacAGAAGAGCTGAATTCAGCTCTTCA**GGCCGGCATGGTCCCAGCCTCCTCGCTGGCGCCGGCTGGGCAACATGCTTCGGCATGGCGAATGGGAC**agagacctgaattcaggtctcaCCTGTCACCGGATGTGCTTTCCGGTCTGATGAGTCCGTGAGGACGAAACAGG

**Figure S7.** crRNA-pRNA cassete sequence with *rrk1* promoter. *rrk1*promoter and leader RNA(2), capital letters; DR, lowercase letters; *Bsp*QI place holder for crRNA, underlined capital letters; HDVR, bold letters; *Bsa*I placer holder for pRNA, underlined lowercase letters; HHR, wave underlined captical letters.

**SUPPLEMENTARY TABLES**

**Table S1.** List of main strains and plasmids used in this study.

| Strains and plasmids | Characteristics | Source/Reference |
| --- | --- | --- |
| **Strains** | | |
| *E.coli* [DH5α](http://www.baidu.com/link?url=_2cqaHxXn_OKbZfgxUEUswAADXyKK09VwEG8ZiomAceKhUSCMxNulzbJr5ie7KjuzMUnC8yk6ZDUVYta7zUVX0HPMFVFV892zvJbyLgOgye2VtsFxWEMMjAyXoFV-l4e4HRZN4eVDAVnQ6A0IwU5-F2YvJzb4AI4NnlG3IT3ba49B0aVXWKRpjLVdZX-QcE97cgbbO3wKRwD22R2Vx5d_q&wd=&eqid=e461002d00003cd1000000035a096904) | *F- eNDA1 glnV44 thi-1 recA1 relA1 gyrA96 deoR nupG*  *Φ80dlacZΔM15 Δ(lacZYA-argF)U169, hsdR17 (rK-mK), λ–* | Takara Biotechnology Co.,Ltd. |
| *S.pombe* FY7652 | *h- leu1-32 ura4-D18* | National Bio Resource Project |
| **Plasmids** | | |
| pBluescript II KS(+) | *E.coli* , *ColE1 ori*, F1 *ori*, Ap ^r^ |  |
| pDUAL-HFF1 | *E.coli* -*S.pombe* shutting vector, *ars1*, *ori*, Ap ^r^ , *ura4* | RIKEN BRC (RDB:6179) |
| pC001 | *E.coli* , *ColE1 ori*, F1 *ori*, Ap ^r^, Cas13a, | Addgene: 79150 |
| pEGFP-N1-FLAG | *E.coli,* eGFP | Addgene: 60360 |
| pmCherry Paxillin | *E.coli,* mCherry | Addgene: 50526 |
| pDUAL-HFF1-eGFP | *FY7652,* eGFP/*nmt1* | This study |
| pDUAL-HFF1-mCherry-eGFP | *FY7652,* mCherry, eGFP/*nmt1* | This study |
| pDUAL-HFF1-mCherry-eGFP-W58X | *FY7652,* mCherry/*nmt1* | This study |
| pDUAL-HFF1-Cas13a | *FY7652,* Cas13a/*nmt1* | This study |
| pDUAL-HFF1-dCas13a-hADAR2d | *FY7652,* dCas13a-hADAR2d/*nmt1* | This study |
| pKS-rrk1-crRNA-backbone | *E.coli,* crRNA-pRNA expression cassete, Amp^r^ | This study |
| pHL414 | *S. pombe,*  Tf1 retrotransposon/*nmt1* | Professor Henry L. Levin’s lab |

**TABLE S2.** Intermediate plasmids for reporter and crRNA/pRNA constructs.

| **Intermediate plasmids** | **Primers** | **Cloning sites** | **Original plasmids** | **Source** |
| --- | --- | --- | --- | --- |
| pKS-mCherry-linker | mCherry-P5  mCherry-linker-P3  mCherry-linker-P5  linker-P3 | *Kpn*I/*Xba*I | pBluescript II KS(+) | This study |
| pKS-mCherry-eGFP | eGFP-P5  eGFP-P3 | *Xba*I/*Bgl*II | pKS-mCherry-linker | This study |
| pKS-mCherry-eGFP-W58X | eGFP-P5  eEGFP-mut-P3  eEGFP-mut-P5  eGFP-P3 | *Xba*I/*Bgl*II | pKS-mCherry-linker | This study |
| pKS-rrk1-crRNA-control | crRNA-eGFP-P5  crRNA-eGFP-P3 | *BspQ*I | pKS-rrk1-crRNA-backbone | This study |
| pKS-rrk1-crRNA-pRNA-seperate | crRNA-eGFP-P5  crRNA-eGFP-P3 | crRNA/*BspQ*I  pRNA/*Bsa*I | pKS-rrk1-crRNA-backbone | This study |
| pKS-rrk1-crRNA-pRNA-fusion | crRNA-L-P5  crRNA-L-P3 | *BspQ*I | pKS-rrk1-crRNA-backbone | This study |
| pKS-rrk1-crRNA-pRNA-6-37 | crRNA-eGFP-fusion-6-37-p5  crRNA-eGFP-fusion-6-37-p3 | *BspQ*I | pKS-rrk1-crRNA-backbone | This study |
| pKS-rrk1-crRNA-pRNA-6-39 | crRNA-eGFP-fusion-6-39-p5  crRNA-eGFP-fusion-6-39-p3 | *BspQ*I | pKS-rrk1-crRNA-backbone | This study |
| pKS-rrk1-crRNA-pRNA-6-49 | crRNA-eGFP-fusion-6-49-p5  crRNA-eGFP-fusion-6-49-p3 | *BspQ*I | pKS-rrk1-crRNA-backbone | This study |
| pKS-rrk1-crRNA-pRNA-6-59 | crRNA-eGFP-fusion-6-59-p5  crRNA-eGFP-fusion-6-59-p3 | *BspQ*I | pKS-rrk1-crRNA-backbone | This study |
| pKS-rrk1-crRNA-pRNA-(-9)-65 | crRNA-eGFP-fusion-(-9)-65-p5  crRNA-eGFP-fusion-(-9)-65-p3 | *BspQ*I | pKS-rrk1-crRNA-backbone | This study |
| pKS-rrk1-crRNA-pRNA-(-5)-65 | crRNA-eGFP-fusion-(-5)-65-p5  crRNA-eGFP-fusion-(-5)-65-p3 | *BspQ*I | pKS-rrk1-crRNA-backbone | This study |
| pKS-rrk1-crRNA-pRNA-(-4)-65 | crRNA-eGFP-fusion-(-4)-65-p5  crRNA-eGFP-fusion-(-4)-65-p3 | *BspQ*I | pKS-rrk1-crRNA-backbone | This study |
| pKS-rrk1-crRNA-pRNA-2-65 | crRNA-eGFP-fusion-(2)-65-p5  crRNA-eGFP-fusion-(2)-65-p3 | *BspQ*I | pKS-rrk1-crRNA-backbone | This study |
| pKS-rrk1-crRNA-pRNA-12-65 | crRNA-eGFP-fusion-(12)-65-p5  crRNA-eGFP-fusion-(12)-65-p3 | *BspQ*I | pKS-rrk1-crRNA-backbone | This study |
| pKS-rrk1-crRNA-pRNA-15-65 | crRNA-eGFP-fusion-(15)-65-p5  crRNA-eGFP-fusion-(15)-65-p3 | *BspQ*I | pKS-rrk1-crRNA-backbone | This study |
| pKS-rrk1-crRNA-pRNA-19-65 | crRNA-eGFP-fusion-(19)-65-p5  crRNA-eGFP-fusion-(19)-65-p3 | *BspQ*I | pKS-rrk1-crRNA-backbone | This study |
| pKS-rrk1-crRNA-pRNA-act1-1566 | crRNA-act1-1566-p5  crRNA-act1-1566-p3 | *BspQ*I | pKS-rrk1-crRNA-backbone | This study |
| pKS-rrk1-crRNA-pRNA-ade6-622 | crRNA-ade6-622-p5  crRNA-ade6-622-p3 | *BspQ*I | pKS-rrk1-crRNA-backbone | This study |
| pKS-rrk1-crRNA-pRNA-ade6-1002 | crRNA-ade6-1003-p5  crRNA-ade6-1003-p3 | *BspQ*I | pKS-rrk1-crRNA-backbone | This study |
| pKS-rrk1-crRNA-pRNA-ade6-1160 | crRNA-ade6-1160-p5  crRNA-ade6-1160-p3 | *BspQ*I | pKS-rrk1-crRNA-backbone | This study |
| pKS-rrk1-crRNA-pRNA-erp5-672 | crRNA-erp5-672-p5  crRNA-erp5-672-p3 | *BspQ*I | pKS-rrk1-crRNA-backbone | This study |
| pKS-rrk1-crRNA-pRNA-mel1-921 | crRNA-mel1-921-p5  crRNA-mel1-921-p3 | *BspQ*I | pKS-rrk1-crRNA-backbone | This study |
| pKS-rrk1-crRNA-pRNA-mug45 | crRNA-mug45-530-p5  crRNA-mug45-530-p3 | *BspQ*I | pKS-rrk1-crRNA-backbone | This study |
| pKS-rrk1-crRNA-pRNA-nmt1-648 | crRNA-nmt1-648-p5  crRNA-nmt1-648-p3 | *BspQ*I | pKS-rrk1-crRNA-backbone | This study |
| pKS-rrk1-crRNA-pRNA-tdh1-79 | crRNA-tdh1-79-p5  crRNA-tdh1-79-p3 | *BspQ*I | pKS-rrk1-crRNA-backbone | This study |
| pKS-rrk1-crRNA-pRNA-(Tf1-835) | crRNA-Tf1-835-p5  crRNA-Tf1-835-p3 | *BspQ*I | pKS-rrk1-crRNA-backbone | This study |
| pKS-rrk1-crRNA-pRNA-(Tf1-1165) | crRNA-Tf1-1165-p5  crRNA-Tf1-1165-p3 | *BspQ*I | pKS-rrk1-crRNA-backbone | This study |
| pKS-rrk1-crRNA-pRNA-(Tf1-control) | crRNA-Tf1-control  crRNA-Tf1-control | *BspQ*I | pKS-rrk1-crRNA-backbone | This study |

**TABLE S3.** Two endogenous genes from *S. pombe* with its transcripts targeted by crRNA constructs.

| **Gene name** | **Gene ID** | **Transcript Size** | **protospacer**  **location** | **Target sequence by crRNA** |
| --- | --- | --- | --- | --- |
| *ade6* | SPCC1322.13 | 1745 | 1130-1194 | ATTTCTGATTCACCTCAAGAATGTGAACGTaGGTATCAGATGCTTCTTGACGTCAAAGATCCTGT |
| *tdh1* | SPBC32F12.11 | 1518 | 49-113 | GCATCGCTTCTGTATAGATCATTCATCCATAGTATTGATTTACACTTGATTCAAAATGGCAATTC |

**TABLE S4.** Constructs for knocking down endogenous gene.

| **Plasmid Name** | **Genes/Promoters** | **crRNA primers** | **Original plasmid** | **Targeting gene** | **Source** |
| --- | --- | --- | --- | --- | --- |
| pDUAL-HFF-Cas13a  -rrk1-crRNA-ade6-1160 | *cas13a*/*nmt1*  crRNA/*rrk1* | crRNA-ade6-1160-p5  crRNA-ade6-1160-p3 | pDUAL-HFF1 | *ade6* | This study |
| pDUAL-HFF-Cas13a  -rrk1-crRNA-tdh1-79 | *cas13a*/*nmt1*  crRNA/*rrk1* | crRNA-tdh1-79-p5  crRNA-tdh1-79-p3 | pDUAL-HFF1 | *tdh1* | This study |

**TABLE S5.** crRNA-pRNA constructs for editing of reporter gene mCherry-eGFP-W58X.

| **Plasmid Name** | **Genes/Promoters** | **crRNA-pRNA primers** | **crRNA-pRNA**  **length (nt)** | **Editing site** | **Source** |
| --- | --- | --- | --- | --- | --- |
| pDUAL-HFF-mCherry-eGFP-W58X  -rrk1-crRNA-control | mCherry-eGFP-W58X /*nmt1*  crRNA/*rrk1* | crRNA-eGFP-P5  crRNA-eGFP-P3 | 28 |  | This study |
| pDUAL-HFF-mCherry-eGFP-W58X  -rrk1-crRNA-pRNA-seperate | mCherry-eGFP-W58X /*nmt1*  crRNA/pRNA/*rrk1* | crRNA-eGFP-P5  crRNA-eGFP-P3  pRNA-HH-P5  pRNA-HH-P3 | 28+37 | 6 | This study |
| pDUAL-HFF-mCherry-eGFP-W58X  -rrk1-crRNA-pRNA-fusion | mCherry-eGFP-W58X /*nmt1*  crRNA-pRNA/*rrk1* | crRNA-L-P5  crRNA-L-P3 | 65 | 6 | This study |
| pDUAL-HFF-mCherry-eGFP-W58X  -rrk1-crRNA-6-37 | mCherry-eGFP-W58X /*nmt1*  crRNA-pRNA(9bp)/*rrk1* | crRNA-eGFP-fusion-6-37-p5  crRNA-eGFP-fusion-6-37-p3 | 28+9 | 6 | This study |
| pDUAL-HFF-mCherry-eGFP-W58X  -rrk1-crRNA-6-39 | mCherry-eGFP-W58X /*nmt1*  crRNA-pRNA(11bp)/*rrk1* | crRNA-eGFP-fusion-6-39-p5  crRNA-eGFP-fusion-6-39-p3 | 28+11 | 6 | This study |
| pDUAL-HFF-mCherry-eGFP-W58X  -rrk1-crRNA-6-49 | mCherry-eGFP-W58X /*nmt1*  crRNA-pRNA(21bp)/*rrk1* | crRNA-eGFP-fusion-6-49-p5  crRNA-eGFP-fusion-6-49-p3 | 28+21 | 6 | This study |
| pDUAL-HFF-mCherry-eGFP-W58X  -rrk1-crRNA-6-59 | mCherry-eGFP-W58X /*nmt1*  crRNA-pRNA(31bp)/*rrk1* | crRNA-eGFP-fusion-6-59-p5  crRNA-eGFP-fusion-6-59-p3 | 28+31 | 6 | This study |
| pDUAL-HFF-mCherry-eGFP-W58X  -rrk1-crRNA-6-65 | mCherry-eGFP-W58X /*nmt1*  crRNA-pRNA(37bp)/*rrk1* | crRNA-eGFP-fusion-6-65-p5  crRNA-eGFP-fusion-6-65-p3 | 28+37 | 6 | This study |
| pDUAL-HFF-mCherry-eGFP-W58X  -rrk1-crRNA-(-9)-65 | mCherry-eGFP-W58X /*nmt1*  crRNA-pRNA(A-9)/*rrk1* | crRNA-eGFP-fusion-(-9)-65-p5  crRNA-eGFP-fusion-(-9)-65-p3 | 28+37 | -9 | This study |
| pDUAL-HFF-mCherry-eGFP-W58X  -rrk1-crRNA-(-5)-65 | mCherry-eGFP-W58X /*nmt1*  crRNA-pRNA(A-5)/*rrk1* | crRNA-eGFP-fusion-(-5)-65-p5  crRNA-eGFP-fusion-(-5)-65-p3 | 28+37 | -9 | This study |
| pDUAL-HFF-mCherry-eGFP-W58X  -rrk1-crRNA-(-4)-65 | mCherry-eGFP-W58X /*nmt1*  crRNA-pRNA(A-4)/*rrk1* | crRNA-eGFP-fusion-(-4)-65-p5  crRNA-eGFP-fusion-(-4)-65-p3 | 28+37 | -9 | This study |
| pDUAL-HFF-mCherry-eGFP-W58X  -rrk1-crRNA-2-65 | mCherry-eGFP-W58X /*nmt1*  crRNA-pRNA(A+2)/*rrk1* | crRNA-eGFP-fusion-(2)-65-p5  crRNA-eGFP-fusion-(2)-65-p3 | 28+37 | 2 | This study |
| pDUAL-HFF-mCherry-eGFP-W58X  -rrk1-crRNA-12-65 | mCherry-eGFP-W58X /*nmt1*  crRNA-pRNA(A+12)/*rrk1* | crRNA-eGFP-fusion-(12)-65-p5  crRNA-eGFP-fusion-(12)-65-p3 | 28+37 | 12 | This study |
| pDUAL-HFF- mCherry-eGFP-W58X  -rrk1-crRNA-15-65 | mCherry-eGFP-W58X /*nmt1*  crRNA-pRNA(A+15)/*rrk1* | crRNA-eGFP-fusion-(15)-65-p5  crRNA-eGFP-fusion-(15)-65-p3 | 28+37 | 15 | This study |
| pDUAL-HFF- mCherry-eGFP-W58X  -rrk1-crRNA-19-65 | mCherry-eGFP-W58X /*nmt1*  crRNA-pRNA(A+19)/*rrk1* | crRNA-eGFP-fusion-(15)-65-p5  crRNA-eGFP-fusion-(15)-65-p3 | 28+37 | 19 | This study |

**TABLE S6.** crRNA-pRNA constructs for editing of endogenous genes.

| Plasmid Name | crRNA-pRNA primers | Promoter | Original plasmid | Targeting gene | Source |
| --- | --- | --- | --- | --- | --- |
| pDUAL-HFF-mCherry-eGFP-W58X  -rrk1-crRNA-act1-1566 | crRNA-act1-1566-p5  crRNA-act1-1566-p3 | *rrk1* | pDUAL-HFF-mCherry-eGFP-W58X | *act1* | This study |
| pDUAL-HFF-mCherry-eGFP-W58X  -rrk1-crRNA-ade6-622 | crRNA-ade6-622-p5  crRNA-ade6-622-p3 | *rrk1* | pDUAL-HFF-mCherry-eGFP-W58X | *ade6* | This study |
| pDUAL-HFF-mCherry-eGFP-W58X  -rrk1-crRNA-ade6-1003 | crRNA-ade6-1003-p5  crRNA-ade6-1003-p3 | *rrk1* | pDUAL-HFF-mCherry-eGFP-W58X | *ade6* | This study |
| pDUAL-HFF-mCherry-eGFP-W58X  -rrk1-crRNA-ade6-1160 | crRNA-ade6-1160-p5  crRNA-ade6-1160-p3 | *rrk1* | pDUAL-HFF-mCherry-eGFP-W58X | *ade6* | This study |
| pDUAL-HFF-mCherry-eGFP-W58X  -rrk1-crRNA-erp5-672 | cRNA-erp5-672-p5  cRNA-erp5-672-p3 | *rrk1* | pDUAL-HFF-mCherry-eGFP-W58X | *erp5* | This study |
| pDUAL-HFF-mCherry-eGFP-W58X  -rrk1-crRNA-mel1-921 | crRNA-mel1-921-p5  crRNA-mel1-921-p3 | *rrk1* | pDUAL-HFF-mCherry-eGFP-W58X | *mel1* | This study |
| pDUAL-HFF-mCherry-eGFP-W58X  -rrk1-crRNA-mug45 | cRNA-mug45-529-p5  cRNA-mug45-529-p3 | *rrk1* | pDUAL-HFF-mCherry-eGFP-W58X | *mug45* | This study |
| pDUAL-HFF-mCherry-eGFP-W58X  -rrk1-crRNA-nmt1-648 | cRNA-nmt1-648-p5  cRNA-nmt1-648-p3 | *rrk1* | pDUAL-HFF-mCherry-eGFP-W58X | *nmt1* | This study |
| pDUAL-HFF-mCherry-eGFP-W58X  -rrk1-crRNA-tdh1-79 | crRNA-tdh1-79-p5  crRNA-tdh1-79-p3 | *rrk1* | pDUAL-HFF-mCherry-eGFP-W58X | *tdh1* | This study |

**TABLE S7. Constucts for editing Tf1 transposon.**

| Plasmid Name | Promoter | Original plasmid | Targeting residue | Source |
| --- | --- | --- | --- | --- |
| pHL414-Tf1-835(G→A) | Tf1/*nmt1* | pHL414 |  | This study |
| pHL414-Tf1-1165(G→A) | Tf1/*nmt1* | pHL414 |  | This study |
| pHL414-Tf1-835(G→A)  -crRNA-pRNA-835 | Tf1/*nmt1*  crRNA-pRNA/*rrk1* | pHL414-Tf1-835(G→A) | Tf1 835 A | This study |
| pHL414-Tf1-835(G→A)  -rrk1-crRNA-pRNA-835-control | Tf1/*nmt1*  crRNA-pRNA/*rrk1* | pHL414-Tf1-835(G→A) |  | This study |
| pHL414-Tf1-1165(G→A)  -rrk1-crRNA-pRNA-1165 | Tf1/*nmt1*  crRNA-pRNA/*rrk1* | pHL414-Tf1-1165(G→A) | Tf1 1165 A | This study |
| pHL414-Tf1-1165(G→A)  -rrk1-crRNA-pRNA-control | Tf1/*nmt1*  crRNA-pRNA/*rrk1* | pHL414-Tf1-1165(G→A) |  | This study |

**References**

1. Komor, A.C., Kim, Y.B., Packer, M.S., Zuris, J.A. and Liu, D.R. (2016) Programmable editing of a target base in genomic DNA without double-stranded DNA cleavage. *Nature*, **533**, 420-424.

2. Jacobs, J.Z., Ciccaglione, K.M., Tournier, V. and Zaratiegui, M. (2014) Implementation of the CRISPR-Cas9 system in fission yeast. *Nat Commun*, **5**, 5344.
